# Effectiveness of dance interventions for falls prevention in older adults: systematic review and meta-analysis

**DOI:** 10.1093/ageing/afae104

**Published:** 2024-05-22

**Authors:** Kimberly Lazo Green, Yang Yang, Ukachukwu Abaraogu, Claire H Eastaugh, Fiona R Beyer, Gill Norman, Chris Todd

**Affiliations:** National Institute for Health and Care Research, Older People and Frailty Policy Research Unit, The University of Manchester, Manchester M13 9PL, UK; Healthy Ageing Research Group, School of Health Sciences, Faculty of Biology, Medicine and Health, The University of Manchester, Manchester M13 9PL, UK; The University of Manchester, Manchester Academic Health Science Centre, Manchester M13 9NQ, UK; The University of Manchester, Manchester Institute for Collaborative Research on Ageing, Manchester M13 9PL, UK; Healthy Ageing Research Group, School of Health Sciences, Faculty of Biology, Medicine and Health, The University of Manchester, Manchester M13 9PL, UK; The University of Manchester, Manchester Academic Health Science Centre, Manchester M13 9NQ, UK; The University of Manchester, Manchester Institute for Collaborative Research on Ageing, Manchester M13 9PL, UK; National Institute for Health and Care Research Applied Research Collaboration—Greater Manchester (NIHR ARC-GM), The University of Manchester, Manchester M13 9PL, UK; National Institute for Health and Care Research, Older People and Frailty Policy Research Unit, The University of Manchester, Manchester M13 9PL, UK; Research Centre for Health, Glasgow Caledonian University, Glasgow G4 0BA, UK; Department of Biological Sciences and Health, University of the West of Scotland, Lanarkshire, Glasgow G72 0LH, UK; National Institute for Health and Care Research Older People and Frailty Policy Research Unit, Newcastle University, Newcastle upon Tyne NE4 5PL, UK; Evidence Synthesis Group/Innovation Observatory, Population Health Sciences Institute, Newcastle University, Newcastle Upon Tyne NE4 5TG, UK; National Institute for Health and Care Research Older People and Frailty Policy Research Unit, Newcastle University, Newcastle upon Tyne NE4 5PL, UK; Evidence Synthesis Group/Innovation Observatory, Population Health Sciences Institute, Newcastle University, Newcastle Upon Tyne NE4 5TG, UK; National Institute for Health and Care Research Applied Research Collaboration—Greater Manchester (NIHR ARC-GM), The University of Manchester, Manchester M13 9PL, UK; Division of Nursing, Midwifery & Social Work, School of Health Sciences, Faculty of Biology, Medicine & Health, University of Manchester, Manchester M13 9PL, UK; National Institute for Health and Care Research, Older People and Frailty Policy Research Unit, The University of Manchester, Manchester M13 9PL, UK; Healthy Ageing Research Group, School of Health Sciences, Faculty of Biology, Medicine and Health, The University of Manchester, Manchester M13 9PL, UK; The University of Manchester, Manchester Academic Health Science Centre, Manchester M13 9NQ, UK; The University of Manchester, Manchester Institute for Collaborative Research on Ageing, Manchester M13 9PL, UK; National Institute for Health and Care Research Applied Research Collaboration—Greater Manchester (NIHR ARC-GM), The University of Manchester, Manchester M13 9PL, UK

**Keywords:** dance interventions, falls, falls prevention, older adults, community-dwelling older adults, systematic review, older people

## Abstract

**Introduction:**

Fall prevention is a global health priority. Strength and balance exercise programmes are effective at reducing falls. Emerging literature suggests dance is an enjoyable and sociable form of exercise. However, there is little evidence that dance reduces fall incidence.

**Methods:**

Systematic review and meta-analysis examining effectiveness and cost-effectiveness of dance for falls prevention in older adults. Five databases were searched with no restrictions on publication date or intervention settings. Risk of bias was assessed using variants of Cochrane Risk of bias tools, Mixed-Methods Appraisal and Drummond checklist as appropriate. Certainty of evidence was assessed using GRADE.

**Results:**

Forty-one studies were included (19 RCTs, 13 quasi-experimental, two mixed-method, seven observational studies, 2,451 participants). Five types of dance interventions were identified: ballroom and Latin dance, dance exercise, cultural dance, dance therapy, and low-impact dance. Meta-analysis was only possible for functional outcome measures: Timed-Up-and-Go (dance versus usual care, mean difference (MD) = 1.36; 95% CI −3.57 to 0.85), Sit-to-Stand (dance versus exercise MD = −0.85; 95% CI −2.64 to 0.93: dance versus education MD = −1.64; 95% CI −4.12 to 0.85), Berg Balance Scale (dance versus usual care MD = 0.61; 95% CI −4.26 to 5.47). There was unexplained variance in effects and no significant differences between intervention and control groups. Overall, certainty of evidence was very low; we are uncertain about the effect of dance interventions in reducing falls.

**Conclusions:**

There is very low certainty evidence for dance as an alternative to strength and balance training if the aim is to prevent falls. No robust evidence on the cost-effectiveness of dance interventions for the prevention of falls was found.

**PROSPERO registration:**

CRD42022382908.

## Key Points

- There is lack of evidence to support dance as an alternative to exercise interventions if the aim is to prevent falls.
- There is very low certainty evidence on the effectiveness of dance interventions in falls prevention.
- Further robust randomised controlled trials are required to evaluate the effectiveness and cost-effectiveness of dance interventions in falls prevention.

## Introduction

A fall is defined as ‘an event which results in a person coming to rest inadvertently on the ground or floor or other lower level’ [1]. Annually, 30% of adults aged 65 years and over fall. It is the second leading cause of unintentional injury or death amongst older people [2] and 5–10% of falls in this group cause injuries (e.g. fractures, head injuries). Falls can also reduce confidence and participation in physical activities [3, 4]. Without effective interventions, injuries are likely to increase with population ageing [5–8]. Intrinsic (e.g. biological, history of falls) and extrinsic (e.g. environmental hazards) risk factors for falls have been identified in order to recommend effective interventions [5, 7, 9]. Falls are a large burden for health expenditure [10], although costs vary internationally.

There is strong evidence that strength and balance exercise programmes reduce falls [8, 11]. The most recent Cochrane review investigating exercise interventions for falls prevention reported an overall falls reduction of 23% [11]. Single exercise programmes like Falls Management Exercise (FaME) [12, 13] and Otago [14, 15] have established effectiveness and are recommended by the United Kingdom (UK) Public Health England as having positive return on investment [16].

Dance is an enjoyable and sociable form of exercise [17–22]. The ProFANE Falls Taxonomy [23] defined dance as a ‘constant movement in a controlled, fluid, repetitive way through all three spatial planes or dimensions (forward and back, side to side, and up and down). . . dance involves a wide range of dynamic movement qualities, speed, and patterns’. Tai Chi (another form of 3D exercise) may prevent falls, but less is known about the effectiveness of dance [11]. A number of reviews have reported physical and mental health benefits of dance [17–22], but programmes do not usually focus on exercise types known to prevent falls, nor are falls their primary outcome. Proxy markers may improve without falls rate or number of fallers reducing [8], so these reviews, whilst suggesting promise of dance interventions, do not address their effectiveness in reducing falls. Information on cost-effectiveness of dance is also lacking in contrast to single factor balance- and strength-focused interventions, such as Otago and FaME [24, 25].

In the UK, resources are being developed to address the predicted increase in falls over the coming years as a result of population ageing exacerbated by reduced physical activity and deconditioning during the COVID-19 pandemic [26, 27]. In this context, our systematic review considers the effectiveness and cost-effectiveness of dance interventions for falls prevention, risk of falls and concerns about falling (also known as fear of falling).

## Methods

Our protocol was registered on PROSPERO (CRD42022382908) [28]; the review is reported according to Preferred Reporting Items for Systematic Review and Meta-Analysis (PRISMA) [29] guidelines.

### Search strategy

The search was designed by an information specialist; the search strategy (Supplementary File A) included terms for age, dance and falls. In December 2022, we searched MEDLINE (Ovid), CINAHL (EBSCO), Epistemonikos (https://www.epistemonikos.org/), CENTRAL (Wiley) and PEDro (https://pedro.org.au/). We did not restrict language, publication date or settings.

### Eligibility criteria

Full inclusion and exclusion criteria are in Supplementary File B. Experimental (randomised controlled trials (RCTs)), quasi-experimental (QE), observational, mixed methods and cost-effectiveness studies investigating dance-based interventions (defined based on the ProFANE Falls Taxonomy [23]) for adults aged ≥50 years were included. We accepted any or no control group but anticipated no intervention, usual care or comparators including alternative dance or exercise. The primary review outcome was falls (falls rate or number of falls). Secondary outcomes were proxy markers for falls (functional assessments, e.g. Berg Balance Score (BBS), Timed-Up-and-Go (TUG), Sit-to-Stand (STS)); risk of falls; concerns about falling; strength; health-related quality of life (HRQoL); and cost effectiveness (where number of falls was reported).

### Study screening and data extraction

Two independent reviewers (KG, UA) screened titles, abstracts and full texts using Rayyan [30]. Disagreements were resolved by involving a third reviewer (CT). One reviewer (KG) used a bespoke data extraction form to record the following: study design, description of participants, interventions and comparators, follow-up period and outcome measures; a second reviewer (YY) checked extraction.

Types of dances were coded iteratively and categorised as: ballroom and Latin (formal social dances with synchronised choreographies, e.g. Argentine Tango), dance-based exercises (low to moderate intensity strength or balance training exercises accompanied by music, e.g. aerobics), cultural dance (dances associated with culture and customs, e.g. folk dance), dance-based therapy (dances used for physiotherapy), and low-impact dance (contemporary dances with low-impact movements, e.g. ballet, tap dance).

### Quality assessment

One reviewer performed quality assessment (KG), and another checked it (YY). Risk of bias was assessed using the Cochrane Risk of Bias (RoB1) tool for RCTs [31]; the Risk of bias In Non-randomised Studies—of Interventions (ROBINS-I) tool for QE studies [32]; the Risk of bias In Non-randomised Studies—of Exposure (ROBINS-E) for observational studies [33]; the Mixed Methods Appraisal Tool (MMAT) for mixed methods studies [34]; and the Drummond checklist for cost-effectiveness studies [35]. For overall rating of the certainty of evidence, we used GRADE (Grading of Recommendations, Assessment, Development and Evaluations) [36].

### Data synthesis

We developed a narrative synthesis structured according to the comparison assessed. Where possible pooled mean differences (MD) with standard deviations (SD) were calculated for RCTs, using random effects model meta-analyses in RevMan (RevMan 5.4). Chi^2^ and I^2^ were used to quantify heterogeneity but not to determine meta-analysis model. We followed Cochrane guidance [37] to interpret heterogeneity: an I^2^ of 0–40% might not be important; 30–60% may represent moderate heterogeneity; 50–75% substantial heterogeneity; and 75–100% considerable heterogeneity. We grouped assessment times into ‘short-term’ (£ 24 weeks), and ‘long-term’ (>24 weeks). Planned subgroup analyses were not possible due to insufficient data.

Sensitivity analyses with a fixed effect model suggested the presence of small study effects and instability and fragility of the confidence intervals in several of the analyses. We have noted where this is the case and have reported the results of all sensitivity analyses in Supplementary File C. In each case where the model results differed substantively the sensitivity (fixed effect) analysis returned a more positive estimate for the intervention effect than the main (random effects) analysis. We adopt a conservative approach in preferring random effects estimates whilst acknowledging their limitations.

Where meta-analysis was impossible, effect direction was synthesised narratively following Synthesis Without Meta-analysis (SWiM) guidelines [38] and using vote-counting methods following Cochrane guidance [39]. Where effect direction varies across multiple outcome measures, we report no clear effect if fewer than 70% of the measures show the same effect direction [40].

## Results

**Figure 1:**
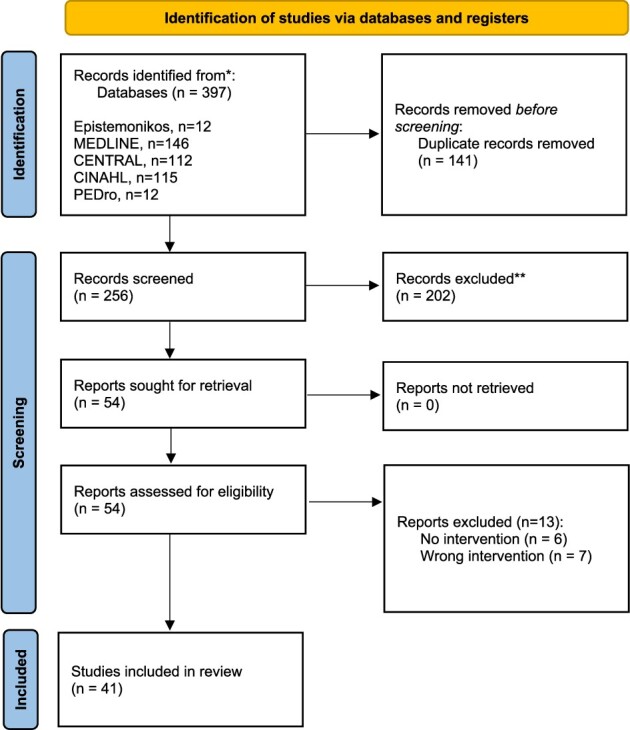
PRISMA diagram.

We identified 397 records. After deduplication, 256 titles, 96 abstracts and 54 full texts were screened. We included 41 studies (Figure 1).

### Study characteristics

Summary of study characteristics are presented in Table 1, with full details in Supplementary File D. There were 19 RCTs [41–59], 13 QE studies (QEs) [60–71], six observational studies [72–77] (including one cost-effectiveness analysis [73]) and three mixed methods studies [78–80]. Populations were older adults who were healthy and active (*N* = 23) [44, 45, 47, 49–55, 58–60, 67, 69, 73, 75, 76, 79–81]; sedentary (*N* = 7) [41–43, 57, 63, 72, 77]; or had conditions such as Parkinson's disease (PD) (*N* = 8) [48, 56, 62, 65, 66, 68, 70, 71], visual impairment (*N* = 2) [64, 74] or dementia (*N* = 1) [61]. Five studies [49, 54, 57, 58, 69] included only women. Duration of dance sessions ranged from 30 minutes to 2 hours weekly, frequency from one to five times weekly and intervention periods from 3 weeks to 14 months [53, 73].

**Table 1:** Summary characteristics of included studies

Author, year	Study design (country)	Population *N*, condition, mean age (SD)	Type of dance	Intervention, comparator and duration
Areeudomwong 2019	RCT (Thailand)	78 sedentary adults. CG: 67.33 (4.04), IG: 66.3 (4.33)	Cultural dance	Thai boxing dance programme Comparator: Education (Falls Prevention booklet) 50 minutes, 3× a week, for 4 weeks
Bennett 2018	RCT (US)	23 sedentary, non-disabled community-dwelling adults. 73.4 (8.4)	Low impact	Line dancing Comparator: Usual care 1 hour, 2× a week, for 8 weeks
Britten 2017	Mixed methods (UK)	38 healthy, community-dwelling adults. 77.3 (8.4)	Low impact	Contemporary dance Comparator: No comparator 90 minutes, 1× a week, for 8 weeks
Buransri 2021	Quasi-experimental (Thailand)	90 healthy, community-dwelling adults. CG: 64.20 (4.5), IG: 63.64 (4.6)	Cultural dance	Traditional Srichiangmai dance Comparator: Exercise (Walking) 30 minutes, 3× a week, for 12 weeks
Charras 2020	Quasi-experimental (France)	23 older adults w/ dementia. 83.47 (5.40)	Dance exercise	Dance exercise Comparator: Usual care 50 minutes, 1× a week, for 24 weeks
da Silva Borges 2014	RCT (Brazil)	59 sedentary adults in long-stay institutions. CG: 67 (7.7), IG: 68 (8.3)	Ballroom and Latin American dance	Ballroom dancing programme Comparator: Usual care 50 minutes, 3× a week, for 12 weeks
de Natale 2017	Quasi-experimental (Italy)	16 adults w/ Parkinson's disease. 67 ± 6.9 CG: 70 (3.16), IG: 66 (9.15)	Ballroom and Latin American dance	Tango Comparator: Exercise (traditional rehabilitation) 60 minutes, 2× a week, for 10 weeks
Federici 2005	RCT (Italy)	40 healthy, community-dwelling adults. CG: 63.5 (3.7), IG: 62.7 (4.1)	Dance exercise	Dance exercise Comparator: Usual care 1 hour, 2× a week, for 3 months
Filar-Mierzwa 2016	Observational (Poland)	24 healthy, sedentary living adults. 66.4	Dance exercise	Dance exercise Comparator: No comparator 45 minutes, 1× a week, for 3 months
Filar-Mierzwa 2021	Quasi-experimental (Poland)	39 healthy, sedentary living adults. CG: 67, IG: 67.45	Dance exercise	Dance exercise Comparator: Exercise (general) 45 minutes, 1× a week, for 3 months
Franco 2020	RCT (Brazil)	82 healthy, community-dwelling adults. 69 (6.6) CG: 70 (6.2), IG: 68.6 (7.2)	Cultural dance	Senior Dance Comparator: Education 1 hour, 2× a week, for 12 weeks
Goldsmith 2021	Cost effectiveness analysis (UK)	1194 older adults. 77	Dance exercise	Dance to Health Comparator: No comparator 2.5 hours, 1× a week, for 56 weeks
Hackney 2013	Observational (US)	13 adults with vision impairment. 86.9 (5.9)	Ballroom and Latin American dance	Adapted tango Comparator: No control group 1.5 hours, 1–2× a week, for 12 weeks
Hackney 2015	Quasi-experimental (US)	32 adults with vision impairment. 79.3 (11) Tango: 84.9 (9), FallProof: 74.8 (11.2)	Ballroom and Latin American dance	Adapted tango Comparator: Exercise (FallProof classes) 1.5 hours, 1–2× a week, for 12 weeks
Hamacher 2016	RCT (Germany)	32 healthy adults. CG: 68.33 (3.17), IG: 66.73 (3.33)	Ballroom and Latin American dance	Dance programme Comparator: Exercise (strength endurance and flexibility training) 90 minutes, 2× a week, for 6 months
Hofgaard 2019	RCT (UK)	27 healthy adults. CG: 74 (4), IG: 75 (5)	Cultural dance	Faroese chain dance programme Comparator: Usual care 30–45 minutes for 6 weeks
Kaewjoho 2020	Observational (Thailand)	61 community-dwelling adults. 72.9 (5.7)	Cultural dance	Thai dance exercise No comparator 50 minutes, 3× a week, for 6 weeks
Kalyani 2020	Quasi-experimental (New Zealand)	33 adults with Parkinson's disease. CG: 66.5 (7.7), IG: 65.24 (11.88)	Dance exercise	Dance for Parkinson’s Disease® (DfPD®) programme Comparator: Usual care 1 hour, 2× a week, for 12 weeks
Krampe 2010	Observational (US)	11 healthy adults (mean age: NR)	Dance therapy	The Lebed Method Dance therapy Comparator: No comparator 45 minutes, 3× a week, for 6 weeks
Kunkel 2017	RCT (UK)	51 adults with Parkinson's disease. CG: 69.7 (6), IG: 71.3 (7.7)	Ballroom and Latin American dance	Mixed dances programme Comparator: Usual care 1 hour, 2× a week, for 10 weeks
Leelapattana 2018	RCT (Thailand)	39 self-ambulatory women. CG: 66.9 (5.6), IG: 66.4 (4.2)	Cultural dance	Thai classical dance exercises Comparator: Exercise (arm-swing exercise) 10 minutes for 12 weeks
Li 2022	RCT (South Korea)	40 healthy adults. CG: 61.75 (1.11), IG: 61.8 (1.23)	Ballroom and Latin American dance	Cha-cha dance training Comparator: Usual care 90 minutes, 3× a week, for 12 weeks
Machacova 2017	RCT (Czech Republic)	189 nursing home residents. CG: 82.88 (8.16), IG: 83.03 (9.10)	Ballroom and Latin American dance	EXDASE (EXercise DAnce for Seniors) Comparator: Usual care 1 hour, 1× a week, for 3 months
McKee 2013	Quasi-experimental (US)	33 adults with idiopathic definite Parkinson's Disease. CG: 74.4 (6.5), IG: 68.4 (7.5)	Ballroom and Latin American dance	Tango Comparator: Education 90 minutes
McKinley 2008	RCT (Canada)	25 healthy, living independently adults. CG: 74.6 (8.4), IG: 78.07 (7.6)	Ballroom and Latin American dance	Argentine Tango dance programme Comparator: Exercise (walking) 2 hours, 2× a week, for 10 weeks
Merom 2016	RCT (Australia)	530 community-dwelling adults. Age > 80: 208 (39%)	Ballroom and Latin American dance	Social dance Comparator: Usual care 1 hour, 2× a week, for 12 months
Noopud 2019	RCT (Thailand)	43 community-dwelling women. CG: 68.29 (5.82), IG: 67.5 (5.39)	Cultural dance	Thai traditional dance Comparator: Usual care 30–60 minutes for 12 weeks
Nur 2022	RCT (Indonesia)	41 community-dwelling adults. CG: 71.6 (10.11), IG: 67.81 (7.731)	Cultural dance	Molong Kopi Comparator: Usual care 15 minutes for 8 weeks
O'Toole 2015	Mixed methods (Ireland)	62 community-dwelling adults. Aged over 70 (*N* = 25; 41.7%	Low impact	Contemporary dance Comparator: No comparator 1× a week for 6 weeks
Pope 2019	Quasi-experimental (US)	163 community-dwelling older adults. CG: 70.7 (6.9); IG: 73.4 (7.7)	Dance therapy	The Lebed Method Dance therapy Comparator: Exercise (Stay Active and Independent for Life (SAIL)) 1 hour, 2–3× a week, for 8–10 weeks
Rawson 2019	RCT (US)	96 adults with idiopathic Parkinson's disease. 78.97 (20.67)	Ballroom and Latin American dance	Argentine tango Comparator: Exercise (stretching and treadmill) 1 hour, 2× a week, for 12 weeks
Rios 2015	RCT (Canada)	33 adults with idiopathic Parkinson's disease. CG: 64.3 (8.1), IG: 63.2 (9.9)	Ballroom and Latin American dance	Argentine Tango Comparator: Usual care 1 hour, 2× a week, for 12 weeks
Rodgrigues-Krause 2018	RCT (Brazil)	30 sedentary women. 65 (5). CG (Stretch): 66 (61–70), Dance: 66 (63–70), Walk: 64 (62–65)	Dance exercise	Structured dancing Comparator: Exercise (walking and stretching) 1 hour, 1–3× a week for 8 weeks
Rodziewicz-Flis 2022	RCT (Poland)	30 community-dwelling women. 73.3 (4.5), CG: 73.4 (5.0), Dance: 72.1 (4.1), Balance: 74.3 (4.6)	Dance exercise	Dance and balance training Comparator: Exercise and usual care 50 minutes, 3× a week, for 12 weeks
Shigematsu 2002	Quasi-experimental (Japan)	38 community-dwelling, healthy independent women. CG: 79.8 (5.0), IG: 78.6 (4.0)	Dance exercise	Dance-based aerobics Comparator: Usual care 1 hour, 3× a week, for 3 months
Sohn 2018	Observational (South Korea)	15 older adults. 72 (5.4)	Ballroom and Latin American dance	Dancesport No comparator 50 minutes, 3× a week, for 15 weeks
Tillmann 2020	Quasi-experimental (Brazil)	20 adults with Parkinson's disease. 66.4 (10.7)	Ballroom and Latin American dance	Brazilian samba Comparator: Usual care 1 hour, 2× a week, for 12 weeks
Vella-Burrows 2021	Mixed methods (UK)	67 older adults	Dance exercise	Dance to Health No comparator 90 minutes, 2–3× a week, for 6 months
Ventura 2016	Quasi-experimental (US)	15 adults with Parkinson's disease. CG: 71.8 (3.6), IG: 70.4 (5.5)	Dance exercise	Dance for Parkinson’s Disease® (DfPD®) programme Comparator: Usual care 1.25 hours, 1× a week, for 5 months
Wang 2021	RCT (China)	44 community-dwelling, healthy independent adults. 64.1 (4.02)	Low impact	Modified tap dance programme Comparator: Education 1 hour, 3× a week, for 12 weeks
Weighart 2020	Quasi-experimental (US)	17 community-dwelling, healthy independent adults. CG: 65.9 (11.9), IG: 73.3 (10.6)	Low impact	Ballet Comparator: Usual care 1 hour, 2× a week, for 10 weeks

CG, control group; IG, intervention group; NR, not reported; RCT, randomised controlled trial; UK, United Kingdom; US, United States.

Seventeen studies assessed ballroom and Latin dances [43, 46, 48, 50–53, 56, 62, 64–66, 68, 70, 71, 74, 77], nine dance-based exercises [44, 57, 58, 61, 63, 69, 72, 73, 79], eight cultural dances [41, 45, 47, 49, 54, 55, 60, 75], two dance-based therapies [67, 76], and five low-impact dances [42, 59, 78, 80, 81].

Comparators were usual activities in 18 studies [42–44, 47, 48, 50, 51, 53–56, 58, 61, 65, 69–71, 81]; exercises (balance and strength training, walking or stretching) in 11 studies [46, 49, 52, 57, 58, 60, 62–64, 67, 68] and education (falls prevention leaflets, promotional materials or seminars) in four studies [41, 45, 59, 66].

Only six studies [43, 53, 56, 66, 73, 75] reported falls (the primary review outcome) (rate of falls or number of falls); only one RCT [53] reported adequate post-intervention data. For secondary outcomes, 35 studies reported various functional tests on dynamic and static balance [41–51, 53–72, 74–76, 81]; most frequently reported were TUG (*N* = 19), STS (*N* = 9) and BBS (*N* = 8). Smaller numbers of studies assessed strength (e.g. arm curl, hand grip, leg strength) [41, 42, 60, 67, 69]; risk of falls [53, 55, 67]; concerns about falling (using the Falls Efficacy Scale–International (FES-I)) [71, 78, 80]; and HRQoL outcomes [48, 53, 61, 64, 66, 70, 71, 78, 79]. One study [73] looked at cost-effectiveness.

### Risk of bias

Detailed assessments of risk of bias are in Supplementary File E.

All of the RCTs had high risk of bias in one or more domains [41–59]; this was driven by performance bias (which was high because it was impossible to blind participants and many personnel to the intervention assignment). Seven RCTs had additional high risk of bias in other domains [41–43, 46, 48, 51, 53, 55]. Detection bias is particularly important where performance bias is inevitable and two trials had high risk of bias [42, 43], with a further eight unclear on this domain [46, 47, 49, 50, 52, 55, 56, 58]. Of 13 QE studies, 4 were at critical risk of bias in almost all key domains [61, 68, 71, 81]; all were likely to have serious to critical overall risk of bias due to bias in selection and classification of participants into interventions. Five observational studies had serious risk of bias consistently across all domains [72, 74–77] and the sixth (cost-effectiveness) study had high risk of bias due to the sample being extrapolated from completers to the whole population [73]; the methods lacked details, with high risk of bias and poor-quality study design. The three mixed methods studies showed high risk of bias in quantitative aspects and poor convergence of outputs from qualitative and quantitative components [78–80].

In GRADE assessments, overall certainty across all comparisons and outcomes is very low due to combinations of risk of bias, imprecision and inconsistency (Table 2).

**Table 2:** Summary of findings

Dance (all types) versus usual care, 18 studies (12 RCTs, 6 QEs)						
Outcomes	Outcome measurement	Follow-up range	Pooled mean difference	Direction of effect^f^	No. of participants (studies)	Certainty of the evidence (GRADE)
Falls		52 weeks	RR 1.23 (1.14, 1.33)		522 (1 RCT)	$\oplus$ ◯◯◯^a^^,^^b^ Very low
		12 weeks		Positive direction of effect. Favours intervention ▲	92 (2 RCTs)	
Functional	Timed up and Go (TUG)	12–20 weeks (short term)	MD −1.36 (−3.57, 0.85)		139 (4 RCTs)	$\oplus$ ◯◯◯^a^^,^^c^^,^^d^ Very low
	Berg Balance Scale (BBS)	8–24 weeks (short term)	MD 0.61 (−4.26, 5.47)		69 (2 RCTs)	
	Various measurements	6–52 weeks		14 of 18 studies (77%) positive direction of effect. Favours intervention ▲	1,183 (12 RCTs and 6 QEs)	
Strength	Various measurements	8–12 weeks		3 of 3 studies (100%). Favours intervention ▲	113 (2 RCTs and 1 QE)	$\oplus$ ◯◯◯^a^^,^^b^ Very low
Quality of life	Various measurements	12–52 weeks		2 of 5 studies (40%) positive direction of effect. No clear effect ◄►	646 (2 RCTs and 3 QEs)	$\oplus$ ◯◯◯^a^^,^^c^ Very low
Concerns about falling	FES-I	20 weeks		Positive direction of effect. Favours intervention ▲	15 (1 QE)	$\oplus$ ◯◯◯^a^^,^^b^ Very low
Risk of falls	Morse Fall, no. of people with no falls risk	8 weeks		Positive direction of effect. Favours intervention ▲	41 (1 RCT)	$\oplus$ ◯◯◯^a^^,^^d^ Very low
	PPA	52 weeks		Negative direction of effect. Does not favour intervention ▼	522 (1 RCT)	
Dance (all types) versus Exercises, 11 studies (5 RCTs, 6 QEs)						
Functional	Sit to stand (STS)	12–14 weeks (short term)	MD−0.85 (−2.64 to 0.93)		64 (2 RCTs)	$\oplus$ ◯◯◯^a^^,^^c^^,^^e^ Very low
	Various measurements	8–24 weeks		6 of 10 studies (60%) positive direction of effect. No clear effect ◄►	470 (3 RCTs and 7 QEs)	
Strength	Various measurements	8–12 weeks		2 of 2 studies (100%) negative direction of effect. Does not favour intervention ▼	229 (2 QEs)	$\oplus$ ◯◯◯^a^^,^^b^^,^^c^ Very low
Quality of life	NEI VFQ-25	16 weeks		Negative direction of effect. Does not favour intervention ▼	32 (1 QE)	$\oplus$ ◯◯◯^a^^,^^b^^,^^c^ Very low
Risk of falls	PPA	8 weeks		1 study (100%) negative direction of effect. Does not favour intervention ▼	139 (1 QEs)	$\oplus$ ◯◯◯^a^^,^^c^ Very low
Dance (all types) versus Education, 4 studies (3 RCTs, 1 QE)						
Falls	No. of falls	12 weeks		No clear effect ◄►	33 (1 QE)	$\oplus$ ◯◯◯^a^^,^^c^ Very low
Functional	Sit to stand (STS)	12 weeks (short term)	MD−1.64 (−4.12 to 0.85)		115 (2 RCTs)	$\oplus$ ◯◯◯^a^^,^^c^^,^^d^ Very low
	Various measurements	12–16 weeks		2 of 3 studies (67%) positive direction of effect. No clear effect ◄►	182 (2 RCTs and 1 QE)	
Strength	Various measurements	16 weeks		Positive direction of effect. Favours intervention ▲	78 (1 RCT)	$\oplus$ ◯◯◯^a^^,^^b^ Very low
Quality of life	Various measurements	12 weeks		Negative direction of effect. Does not favour intervention ▼	33 (1 QE)	$\oplus$ ◯◯◯^a^^,^^b^^,^^c^ Very low
Dance with no comparator, 9 studies (6 observational, 2 mixed methods, 1 cost-effectiveness analysis)						
Falls	No. of falls	24–56 weeks		2 of 2 studies (100%). Favours intervention ▲	307 (1 Obs, 1 CEA)	$\oplus$ ◯◯◯^a^ Very low
Functional	Various measurements	6–16 weeks		6 of 6 studies (100%). Favours intervention ▲	141 (6 Obs)	$\oplus$ ◯◯◯^a^^,^^b^^,^^e^ Very low
Quality of life	Various measurements	6–24 weeks		2 of 2 studies (100%). Favours intervention ▲	78 (2 MM)	$\oplus$ ◯◯◯^a^^,^^b^ Very low
Concerns about falling	FES-I	6–8 weeks		2 of 2 studies (100%). Favours intervention ▲	55 (2 MM)	$\oplus$ ◯◯◯^a^^,^^b^ Very low
Cost-effectiveness	Cost savings	56 weeks		Positive direction of effect. Favours intervention ▲	246 (1 CEA)	$\oplus$ ◯◯◯^a^ Very low

^a^High risk of bias in multiple domains,

^b^Small number of participants,

^c^Wide confidence intervals that include both benefit and harm as well as no effect,

^d^Non-overlap in confidence intervals,

^e^Point estimates vary widely across studies,

^f^Excluding studies that were pooled in meta-analyses.

BBS, Berg Balance Scale; CEA, cost-effectiveness analysis; FES-I, Falls Efficacy Scale–International; FRT M–CTSIB, Fall Risk Test in Modified Clinical Test of Sensory Interaction in Balance; MD, mean difference; NEI VFQ-25, National Eye Institute Visual Function Questionnaire; PPA, Physiological Performance Assessment; Obs, observational study; RCT, randomised controlled trial; RR, risk ratio; TUG, Timed up and Go.

### Effectiveness of interventions

Meta-analysis was impossible for most outcomes; we provide a narrative synthesis based on effect directions and evidence certainty. Table 3 shows the effect direction plot; all calculated effect estimates are in Supplementary File F.

**Table 3:** Direction of effect of included studies

Dance (all types) versus Usual care, 18 studies (12 RCTs, 6 QEs)										
Author, year	Study design	No. of participants	Time point	Falls	Functional	Strength	Quality of life	Concerns about falling	Risk of falls	Cost-effectiveness
Bennett 2018	RCT	23	8 weeks		▲	▲				
Charras 2020	QE	42	12 weeks		▼		▼			
Da Silva Borges 2014	RCT	59	12 weeks	▲	▲					
Federici 2005	RCT	40	12 weeks		▲					
Hofgaard 2019	RCT	25	6 weeks		▲					
Kalyani 2020	QE	33	12 weeks		▲					
Kunkel 2017	RCT	46	24 weeks		▼		▼			
Li 2022	RCT	40	12 weeks		▲					
Machacova 2017	RCT	189	12 weeks		▲	▲				
Merom 2018	RCT	522	52 weeks	▼	▲		▼		▼	
Noopud 2019	RCT	43	12 weeks		▲					
Nur 2022	RCT	41	8 weeks		▼				▲	
Rios 2015	RCT	33	12 weeks	▲	▲					
Rodziewicz-Flis 2022	RCT	20	12 weeks		▲					
Shigematsu 2002	QE	38	12 weeks		▲	▲				
Tillman 2020	QE	20	12 weeks		▲		▲			
Ventura 2016	QE	15	20 weeks		▲		▲	▲		
Weighart 2020	QE	17	10 weeks		▼					
Overall direction of effect				◄►	▲	▲	◄►	▲	◄►	
Dance (all types) versus Exercises, 11 studies (5 RCTs, 6 QEs)										
Buransri 2021	QE	90	12 weeks		▲	▼				
De Natale 2017	QE	16	18 weeks		▲					
Filar-Mierzwa 2021	QE	48	12 weeks		▼					
Hackney 2015	QE	32	16 weeks		▲		▼			
Hamacher 2016	RCT	32	24 weeks		▲					
Leelapattana 2018	RCT	39	12 weeks		▲					
McKinley 2008	RCT	25	14 weeks		▲					
Pope 2019	QE	139	8 weeks		▼	▼			▼	
Rawson 2019	QE	70	12 weeks		▼					
Rodrigues-Krause 2018	RCT	20	8 weeks		▼					
Rodziewicz-Flis 2022	RCT	20	12 weeks		▲					
Overall direction of effect					◄►	▼	▼		▼	
Dance (all types) versus Education, 4 studies (3 RCTs, 1 QE)										
Areeudomwong 2019	RCT	78	16 weeks		▲	▲				
Franco 2020	RCT	71	16 weeks		▲					
McKee 2013	QE	33	12 weeks	◄►	▼		▼			
Wang 2021	RCT	44	12 weeks		▲					
Overall direction of effect				◄►	◄►	▲	▼			
Dance with no comparator, 9 studies (6 obs, 2 MM, 1 CEA)										
Britten 2017	MM	20	8 weeks		▲			▲		
Filar-Mierzwa 2016	Obs	24	12 weeks		▼					
Goldsmith 2021	CEA	246	56 weeks	▲						▲
Hackney 2013	Obs	13	16 weeks		▲					
Kaewjoho 2020	Obs	61	24 weeks	▲	▲					
Krampe 2010	Obs	11	6 weeks		▲					
Otoole 2015	MM	35	6 weeks				▲	▲		
Sohn 2018	Obs	15	15 weeks		▲					
Vella-Burrows 2021	MM	43	24 weeks				▲			
Overall direction of effect				▲	▲		▲	▲		▲

▲ Positive direction of effect; ▼ Negative direction of effect; ◄► No clear effect (<70% of studies)

CEA, cost-effectiveness analysis; MM, mixed methods; Obs, observational study; QE, quasi-experimental study; RCT: randomised controlled trial.

### Dance (all types) versus usual care

There were 12 RCTs [42–44, 47, 48, 50, 51, 53–56, 58] and six QEs [61, 65, 69–71, 81] with 1,246 participants in total, in which control groups did not receive an intervention and carried on with their usual routine.

#### Falls

Three studies recorded falls (3 RCTs, *N* = 614) [43, 53, 56]. Only one RCT reported post-intervention data [53] showing a 23% increased risk in falling (RR = 1.23, 95% CI 1.14–1.33, *N* = 522, 1 RCT) for community-dwelling older adults in a ballroom and Latin dance programme (follow-up of 52 weeks) [53]. The two other RCTs did not report adequate data for effect size calculation [43, 56]. One (*N* = 59) reported fewer falls by sedentary older adults in long-term facilities in the ballroom and Latin dance group (*P* < 0.0001) [43]. The other (*N* = 33) reported number of falls as the most common adverse event; 11% of older adults with PD experienced falls whilst participating in an adapted Argentine tango dance programme [56]. Evidence was downgraded for risk of bias and imprecision.

#### Functional measures

All 18 studies assessed at least one functional outcome (12 RCTs, 6 QEs, *N* = 1,246). For both TUG (MD = −1.36, 95% CI −3.57 to 0.85, I^2^ = 98%, *N* = 139, 4 RCTs) [44, 48, 56, 58] (Figure 1a) and BBS (MD = 0.61, 95% CI −4.26 to 5.47, I^2^ = 70%, *N* = 69, 2 RCTs) [42, 48] (Figure 2.2) there was substantial heterogeneity and no clear differences between groups (follow-up period of 8–24 weeks). Sensitivity analysis using a fixed effect model showed a positive effect of dance (see Supplementary File E); this supports our assessment that there is serious imprecision of the effect estimate. Across all functional measures in the 18 studies, 10 RCTs [42–44, 47, 50, 51, 53, 54, 56, 58] and four QEs [65, 69–71] reported a positive effect direction for dance; there was heterogeneity in interventions, populations and outcome measures. Evidence was downgraded for imprecision, inconsistency and risk of bias.

**Figure 2:**
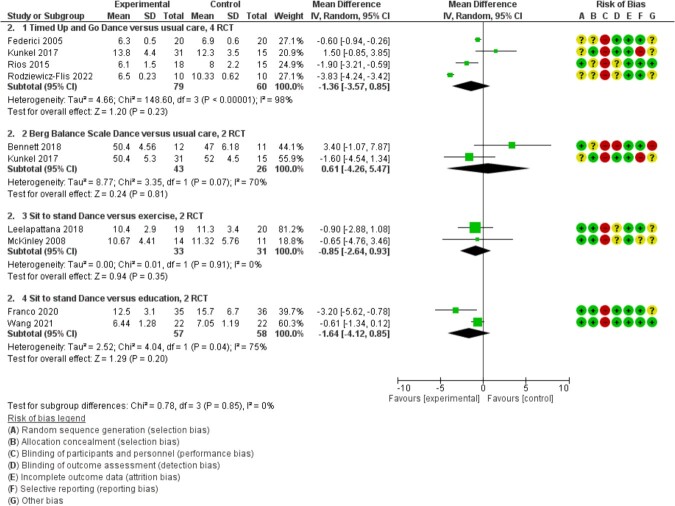
Pooled mean differences. 2.1. Timed Up and Go Dance versus usual care, 4 RCTs. 2.2. Berg Balance Scale Dance versus usual care, 2 RCTs. 2.3. Sit to stand Dance versus exercise, 2 RCTs. 2.4. Sit to stand Dance versus education, 2 RCTs.

#### Strength

Three studies assessed some measure of strength (2 RCTs, 1 QE, *N* = 113) [42, 51, 69]. Studies were small, with short follow-up (8–12 weeks). All reported a positive effect direction favouring dance. Evidence was downgraded for imprecision and risk of bias.

#### Quality-of-life

Quality-of-life outcomes were reported by five studies (2 RCTs, 3 QEs, *N* = 646) [48, 53, 61, 70, 71]. Of these five studies [48, 53, 61, 70, 71], two (both in people with PD) reported positive effect directions favouring dance [70, 71]. Evidence was downgraded for imprecision and risk of bias.

#### Concerns about falling

Only one study (1 QE, *N* = 15) reported concerns about falling [71]. After participating in a dance exercise programme, older adults with PD reported a positive effect on FES-I scores versus usual care [71]. Evidence was downgraded for imprecision and risk of bias.

#### Risk of falls

Risk of falls were assessed by two studies (2 RCTs, *N* = 563) [53, 55]. One small study reported positive effect direction, where the number of older adults not at risk of falls increased (*P* < 0.05) after participating in an 8-week cultural dance programme (*N* = 41) [55]. A larger study (*N* = 522) found significant improvements on Physiological Performance Assessment (PPA) scores after a 52-week ballroom and Latin dance intervention [53]. Evidence was downgraded for inconsistency and risk of bias.

### Dance (all types) versus exercises

Five RCTs [46, 49, 52, 57, 58] and six QEs [60, 62–64, 67, 68] compared dance interventions with various types of exercises (*N* = 531). None of the studies recorded falls or concerns about falling.

#### Functional measures

All 11 studies assessed functional measures (5 RCTs, 6 QEs, *N* = 531). There was no clear difference between the groups on STS (MD = −0.85, 95% CI −2.64 to 0.93, I^2^ = 0%, *n* = 62, 2 RCTs) [49, 52] (Figure 2.3). Six studies (three RCTs [46, 49] and three QEs [60, 62, 64]) reported positive directions of effect favouring dance on functional tests over follow-up periods of 8–24 weeks. Evidence was downgraded for risk of bias, inconsistency and imprecision.

#### Strength

For strength, only two studies examined dance versus exercises (2 QEs, *N* = 229) [60, 67]. Both studies showed negative effect directions for dance compared to exercise on various measurements of arm and leg strength, with confidence intervals including both benefit and harm [60, 67]. Evidence was downgraded for imprecision and high risk of bias.

#### Quality of life

Only one study measured quality of life (1 QE, *N* = 32) [64]. An adaptive Tango class was compared to a balance and mobility programme for older adults with vison impairment [64]. The results show a negative effect direction for dance on the vision-related QOL scores on 1-month post-test. Evidence was downgraded for imprecision and high risk of bias.

#### Risk of falls

Risk of falls was assessed by only one study (1 QE, *N* = 139) [67]. The study reported negative effect direction [67]; the dance group had significantly higher falls risk scores compared to the exercise comparator group. Evidence was downgraded for imprecision and risk of bias.

### Dance (all types) versus education

Three RCTs [41, 45, 59] and one QE [66] compared dance interventions with education (*N* = 226). None of the studies assessed concerns about falling or risk of falls.

#### Falls

Only one study reported falls (1 QE, *N* = 33) [66]. It recorded number of fallers as adverse events whilst participating in an adapted Tango intervention for older adults with PD. Two non-injurious falls were recorded in the dance group and none in the control group; the effect direction was unclear because of the small number of participants and evidence was downgraded for risk of bias and imprecision.

#### Functional outcomes

All four studies assessed functional measures (3 RCTs, 1 QE, *N* = 226). There was no clear difference between the groups in STS (MD = −1.64, 95% CI −4.12 to 0.85, I^2^ = 75%, *N* = 115, 2 RCTs) [45, 59] (Figure 2.4). When sensitivity analysis was performed using a fixed effect, the model showed a positive effect of dance (see Supplementary File E); this supports our assessment that there is serious imprecision of the effect estimate. Of the four studies [41, 45, 59, 66], two reported positive directions of effect favouring dance [41, 45]. Evidence was downgraded for risk of bias and imprecision.

#### Strength

One RCT (1 RCT, *N* = 78) found positive improvements in hip, ankle and knee strength after sedentary older adults participated in a cultural dance programme versus the education group who received a Falls Prevention booklet [41]. Evidence was downgraded for imprecision and risk of bias.

#### Quality of life

One study for older adults with Parkinson’s disease (1 QE, *N* = 33) [66] found a negative effect direction of the dance intervention on HRQoL outcomes compared to an exercise control. Evidence was downgraded for imprecision and risk of bias.

### Dance (all types) with no control

Nine studies had no comparator (*N* = 468) [72–80]. Post-intervention falls were recorded in two studies [73, 75]. For a cultural dance programme, there was a reduction in single falls incidence from 20/61 (33%) to 4/61 (6%) after 24 weeks [75]. A 56-week dance exercise programme reported a 52% reduction in falls incidence, but data were reported for only a subset of the sample (*N* = 246 from 1,194) [73]. The overall certainty of evidence is very low due to the study designs and serious risk of bias across all domains.

For functional tests, quality of life, and concerns about falling, all nine studies reported an overall positive impact of dance interventions on outcomes recorded [72, 74–80]. One study reported a negative although statistically non-significant effect direction for risk of falls, after dance exercise participation [72]. No studies assessed strength. One study, related to a UK-based dance intervention called ‘Dance to Health’, reported cost-effectiveness [73]. The authors claimed potential cost savings of £196 million based on admissions to Accident and Emergency services; this is very low certainty evidence due to the study design and poor quality and follow-up of only completers.

## Discussion

### Summary of evidence

Echoing the results of a recent Cochrane review on interventions on falls prevention [11] and in line with World Guidelines for Falls Prevention and Management [8] conclusions, this review finds that there is weak and inconclusive evidence about dance interventions and their effectiveness in falls prevention.

We found few studies which reported falls. Fewer than half of studies were RCTs, and these showed methodological limitations. Only six studies presented information about number of falls after participating in dance interventions [43, 53, 56, 66, 73, 75]; only three were RCTs and only one presented adequate data to calculate between-group differences [53]. Effectiveness of falls prevention programmes is best assessed when a rigorous RCT design is used, numbers of falls and people who fall are assessed before and after the intervention in each group and when intention to treat analysis is used [1].

We are aware of two recent studies published after our search dates [82, 83]. These were both very small and underpowered for the primary outcome of falls rates and would not modify our finding that there is insufficient evidence for falls prevention in this review. They did suggest that dance interventions may modify fall risk factors, and provide improvements in physical activity, but due to their size, they are unlikely to substantively impact our findings for any outcome.

Across the secondary outcomes of functional measures, strength, quality of life, concerns about falling and risk of falls, there were no consistent effects of dance compared to controls and active comparators. Heterogeneity was only partially explained by variability in interventions and populations and contributed to the identification by GRADE assessments of multiple issues with imprecision and inconsistency. The two recent papers identified would not change these overall GRADE assessments.

Together with risk of bias, this made all evidence very low certainty; we are uncertain as to the effects of dance interventions for fall prevention relative to any comparator.

### Gaps and limitations

We used rigorous systematic review methods throughout and conducted meta-analyses where possible. However, our synthesis is limited by a number of factors.

Most studies used various proxy measures (e.g. functional measures, strength, quality of life, risk of falls, and concerns about falling) as indirect indicators for falls. There were conflicting findings between randomised and non-randomised studies across assessed outcome measures, which varied widely across all studies, with meta-analyses only possible for some outcome measures (TUG, STS and BBS). This is echoed by some more recent evidence on dance interventions where functional measures on balance and strength were used as proxy measures for falls [82, 83].

Follow-up periods were mostly short term (under 24 weeks), although falls prevention interventions likely require longer follow-up periods (over 12 months) because of delayed effects of compliance [1]. Of 41 included studies, only two (one RCT) had follow-up of over a year [53, 73]. Small sample sizes also limited studies’ ability to identify intervention effects.

There was considerable heterogeneity of dance interventions, settings and populations. Older adults with conditions such as PD and dementia have specific risk factors that make them more prone to falls and falls-related injuries. Whilst tailoring falls prevention, interventions based on these modifiable risk factors are recommended [8]; none of the included studies combined dance with individualised exercise programmes that include tailored balance and resistance training for people with PD and dementia [48, 56, 61, 62, 65, 66, 68, 70, 71]. Such combined programmes may be an appropriate development given the lack of evidence for dance interventions in these groups.

Cost-effectiveness of interventions is best evaluated using intention-to-treat RCT data, with robust and triangulated reporting methods for outcomes to populate an economic evaluation [35]. We found only a single observational study [73] with high risk of bias that is best considered as indicating need for further research. Further RCTs would likely be required for rigorous cost-effectiveness evaluation.

Only 11 included studies used exercise and physical activity comparators [46, 49, 52, 57, 58, 60, 62–64, 67, 68]. Amongst these, only three were balance and strength training exercises, such as FallProof balance and mobility programme [64], Stay Active and Independent for Life (SAIL) multifactorial exercise [67] and strength endurance and flexibility training [46]. Much of the evidence may therefore be indirectly relevant to current standards of care. Head-to-head RCTs of dance interventions versus interventions with known effectiveness, such as FaME or Otago exercises, may be warranted.

### Implications for policy and practice

Dance is an enjoyable exercise that provides physical and mental health benefits for older adults [17, 20]. However, it is unknown whether dance is a safe and effective alternative to strength and balance training programmes for reducing falls, although Tai Chi, another 3D exercise, may be [11]. The World Guidelines for Falls Prevention and Management conclude that evidence for effectiveness of dance for falls prevention is very low certainty [8] and our findings align with this, as well as the Cochrane review [11]. There is, therefore, no basis to prioritise dance over exercise programmes with known effectiveness for people at any risk level. Older people at low falls risk may potentially benefit from dance as part of a general healthy lifestyle, but it should not be offered as a falls prevention activity outside of research contexts. In line with the guidelines [8], older adults at intermediate and higher levels of risk should be offered targeted exercise or physiotherapist referral; those at high risk for falls should be offered a multifactorial falls risk assessment to inform individualised tailored interventions.

## Conclusion

Robust evidence for the effectiveness and cost-effectiveness of dance interventions in falls prevention is lacking. Dance may provide benefits to older people who take part but has very low certainty evidence and is not supported as an alternative to structured exercise interventions.

## Supplementary Material

Supplementary_material_afae104

## Data Availability

This review is based on previously published studies. The data that support the findings of this study are available from the corresponding author upon reasonable request.
