# Supplementary material for: Effectiveness of dance interventions for falls prevention in older adults: systematic review and meta-analysis
Source: Age Ageing. 2024 May 22;53(5):afae104. doi: 10.1093/ageing/afae104 (PMC11110915; doi:10.1093/ageing/afae104)
Supplement: Supplementary_material_afae104 [file supplementary_material_afae104.docx]

| **Section and Topic** | **Item #** | **Checklist item** | **Location where item is reported** |
| --- | --- | --- | --- |
| **TITLE** | | |  |
| Title | 1 | Identify the report as a systematic review. | 1 |
| **ABSTRACT** | | |  |
| Abstract | 2 | See the PRISMA 2020 for Abstracts checklist. | 1 |
| **INTRODUCTION** | | |  |
| Rationale | 3 | Describe the rationale for the review in the context of existing knowledge. | 2 |
| Objectives | 4 | Provide an explicit statement of the objective(s) or question(s) the review addresses. | 3 |
| **METHODS** | | |  |
| Eligibility criteria | 5 | Specify the inclusion and exclusion criteria for the review and how studies were grouped for the syntheses. | 3 |
| Information sources | 6 | Specify all databases, registers, websites, organisations, reference lists and other sources searched or consulted to identify studies. Specify the date when each source was last searched or consulted. | 3 |
| Search strategy | 7 | Present the full search strategies for all databases, registers and websites, including any filters and limits used. | 3 |
| Selection process | 8 | Specify the methods used to decide whether a study met the inclusion criteria of the review, including how many reviewers screened each record and each report retrieved, whether they worked independently, and if applicable, details of automation tools used in the process. | 4 |
| Data collection process | 9 | Specify the methods used to collect data from reports, including how many reviewers collected data from each report, whether they worked independently, any processes for obtaining or confirming data from study investigators, and if applicable, details of automation tools used in the process. | 4 |
| Data items | 10a | List and define all outcomes for which data were sought. Specify whether all results that were compatible with each outcome domain in each study were sought (e.g. for all measures, time points, analyses), and if not, the methods used to decide which results to collect. | 4 |
|  | 10b | List and define all other variables for which data were sought (e.g. participant and intervention characteristics, funding sources). Describe any assumptions made about any missing or unclear information. | 4 |
| Study risk of bias assessment | 11 | Specify the methods used to assess risk of bias in the included studies, including details of the tool(s) used, how many reviewers assessed each study and whether they worked independently, and if applicable, details of automation tools used in the process. | 4 |
| Effect measures | 12 | Specify for each outcome the effect measure(s) (e.g. risk ratio, mean difference) used in the synthesis or presentation of results. | 4-5 |
| Synthesis methods | 13a | Describe the processes used to decide which studies were eligible for each synthesis (e.g. tabulating the study intervention characteristics and comparing against the planned groups for each synthesis (item #5)). | 4-5 |
|  | 13b | Describe any methods required to prepare the data for presentation or synthesis, such as handling of missing summary statistics, or data conversions. | 4-5 |
|  | 13c | Describe any methods used to tabulate or visually display results of individual studies and syntheses. | 4-5 |
|  | 13d | Describe any methods used to synthesize results and provide a rationale for the choice(s). If meta-analysis was performed, describe the model(s), method(s) to identify the presence and extent of statistical heterogeneity, and software package(s) used. | 4-5 |
|  | 13e | Describe any methods used to explore possible causes of heterogeneity among study results (e.g. subgroup analysis, meta-regression). | 4-5 |
|  | 13f | Describe any sensitivity analyses conducted to assess robustness of the synthesized results. | 4-5 |
| Reporting bias assessment | 14 | Describe any methods used to assess risk of bias due to missing results in a synthesis (arising from reporting biases). | 4 |
| Certainty assessment | 15 | Describe any methods used to assess certainty (or confidence) in the body of evidence for an outcome. | 4 |
| **RESULTS** | | |  |
| Study selection | 16a | Describe the results of the search and selection process, from the number of records identified in the search to the number of studies included in the review, ideally using a flow diagram. | Figure 1 |
|  | 16b | Cite studies that might appear to meet the inclusion criteria, but which were excluded, and explain why they were excluded. | NA |
| Study characteristics | 17 | Cite each included study and present its characteristics. | 5 |
| Risk of bias in studies | 18 | Present assessments of risk of bias for each included study. | 6, Appendix |
| Results of individual studies | 19 | For all outcomes, present, for each study: (a) summary statistics for each group (where appropriate) and (b) an effect estimate and its precision (e.g. confidence/credible interval), ideally using structured tables or plots. | Table 1, Appendix |
| Results of syntheses | 20a | For each synthesis, briefly summarise the characteristics and risk of bias among contributing studies. | 7-10 |
|  | 20b | Present results of all statistical syntheses conducted. If meta-analysis was done, present for each the summary estimate and its precision (e.g. confidence/credible interval) and measures of statistical heterogeneity. If comparing groups, describe the direction of the effect. | 7-10 |
|  | 20c | Present results of all investigations of possible causes of heterogeneity among study results. | 7-10 |
|  | 20d | Present results of all sensitivity analyses conducted to assess the robustness of the synthesized results. | 7-10 |
| Reporting biases | 21 | Present assessments of risk of bias due to missing results (arising from reporting biases) for each synthesis assessed. | 7-10 |
| Certainty of evidence | 22 | Present assessments of certainty (or confidence) in the body of evidence for each outcome assessed. | 7-10 |
| **DISCUSSION** | | |  |
| Discussion | 23a | Provide a general interpretation of the results in the context of other evidence. | 11 |
|  | 23b | Discuss any limitations of the evidence included in the review. | 11 |
|  | 23c | Discuss any limitations of the review processes used. | 11 |
|  | 23d | Discuss implications of the results for practice, policy, and future research. | 12 |
| **OTHER INFORMATION** | | |  |
| Registration and protocol | 24a | Provide registration information for the review, including register name and registration number, or state that the review was not registered. | 3 |
|  | 24b | Indicate where the review protocol can be accessed, or state that a protocol was not prepared. | 3 |
|  | 24c | Describe and explain any amendments to information provided at registration or in the protocol. | 3 |
| Support | 25 | Describe sources of financial or non-financial support for the review, and the role of the funders or sponsors in the review. | Title page |
| Competing interests | 26 | Declare any competing interests of review authors. | Title page |
| Availability of data, code and other materials | 27 | Report which of the following are publicly available and where they can be found: template data collection forms; data extracted from included studies; data used for all analyses; analytic code; any other materials used in the review. | Title page |

*From:*  Page MJ, McKenzie JE, Bossuyt PM, Boutron I, Hoffmann TC, Mulrow CD, et al. The PRISMA 2020 statement: an updated guideline for reporting systematic reviews. BMJ 2021;372:n71. doi: 10.1136/bmj.n71

For more information, visit: <http://www.prisma-statement.org/>

**Appendix**

**Supplementary File A: Search Strategy**

**Search strategy**

All searches carried out 02 December 2022

**Epistemonikos**

(title:((title:(((elder* OR eldest OR "old* age*" OR senior* OR aged OR geriatri*) OR (older AND (person* OR people OR woman OR women OR female* OR man OR men OR male* OR adult* OR patient* OR population* OR subject*)) OR ("over 50*" OR "over 55*" OR "over 60*" OR "over 65*" OR "over 70*" OR over 75*" OR "over 80*" OR "over 85*" OR "over 90*" OR "over 95*" OR "over 100*") OR "post-menopaus*" OR postmenopaus* OR menopaus* OR (quinquagenarian* OR sexagenarian* OR septuagenarian* OR octogenarian* OR nonagenarian* OR centenarian* OR supercentenarian* OR senium))) OR abstract:(((elder* OR eldest OR "old* age*" OR senior* OR aged OR geriatri*) OR (older AND (person* OR people OR woman OR women OR female* OR man OR men OR male* OR adult* OR patient* OR population* OR subject*)) OR ("over 50*" OR "over 55*" OR "over 60*" OR "over 65*" OR "over 70*" OR over 75*" OR "over 80*" OR "over 85*" OR "over 90*" OR "over 95*" OR "over 100*") OR "post-menopaus*" OR postmenopaus* OR menopaus* OR (quinquagenarian* OR sexagenarian* OR septuagenarian* OR octogenarian* OR nonagenarian* OR centenarian* OR supercentenarian* OR senium)))) AND

(title:(((dance OR dancing) OR ("dance movement" OR "dance exercise" OR "danc* therapy"))) OR abstract:(((dance OR dancing) OR ("dance movement" OR "dance exercise" OR "danc* therapy")))) AND

(title:(((fall OR falls OR falling OR faller) OR ("accident prevention" OR "accidental falls") OR ("fall risk" OR "risk of falling"))) OR abstract:(((fall OR falls OR falling OR faller) OR ("accident prevention" OR "accidental falls") OR ("fall risk" OR "risk of falling")))))

[Filters: protocol=no, classification=systematic-review]

**MEDLINE**

Database(s): **Ovid MEDLINE(R) and Epub Ahead of Print, In-Process, In-Data-Review & Other Non-Indexed Citations, Daily and Versions**1946 to December 01, 2022

Search Strategy:

| **#** | **Searches** | **Results** |
| --- | --- | --- |
| 1 | exp Aged/ | 3425630 |
| 2 | geriatrics/ | 31212 |
| 3 | middle aged/ | 4700938 |
| 4 | (elder or eldest or "old* age*" or senior* or aged or geriatri*).ti,ab,kw. | 880249 |
| 5 | (older and (person* or people or woman or women or female* or man or men or male* or adult* or patient* or population* or subject*)).ti,ab,kw. | 475013 |
| 6 | ("over 50*" or "over 55*" or "over 60*" or "over 65*" or "over 70*" or "over 75*" or "over 80*" or "over 85*" or "over 90*" or "over 95*" or "over 100*").ti,ab,kw. | 130167 |
| 7 | (quinquagenarian* or sexagenarian* or septuagenarian* or octogenarian* or nonagenarian* or centenarian* or supercentenarian* or senium).ti,ab,kw. | 7863 |
| 8 | Menopause/ | 29638 |
| 9 | ("post-menopaus*" or postmenopaus* or menopaus*).ti,ab,kw. | 100082 |
| 10 | or/1-9 | 6215371 |
| 11 | Dancing/ | 3414 |
| 12 | Dance Therapy/ | 439 |
| 13 | (dance or dancing).ti,ab,kw,kf. | 6980 |
| 14 | ((dance adj3 exercise*) or (dance adj3 movement) or (dance adj3 therapy)).ti,ab,kw. | 643 |
| 15 | or/11-14 | 8224 |
| 16 | Accident Prevention/ | 9262 |
| 17 | Accidental Falls/ | 27724 |
| 18 | fall*.ti,ab,kw. | 241023 |
| 19 | ((accident* adj3 fall*) or (accident* adj3 prevention)).ti,ab,kw. | 5276 |
| 20 | ((fall* adj3 reduc*) or (fall* adj3 risk)).ti,ab,kw. | 16264 |
| 21 | or/16-20 | 257637 |
| 22 | 10 and 15 and 21 | 146 |

**CENTRAL**

| **ID** | **Search** | **Hits** |
| --- | --- | --- |
| #1 | MeSH descriptor: [Aged] explode all trees | 221552 |
| #2 | MeSH descriptor: [Geriatrics] this term only | 213 |
| #3 | MeSH descriptor: [Middle Aged] this term only | 334423 |
| #4 | ((elder or eldest or "old* age*" or senior* or aged or geriatri*)):ti,ab,kw | 601325 |
| #5 | ((older and (person* or people or woman or women or female* or man or men or male* or adult* or patient* or population* or subject*))):ti,ab,kw | 65581 |
| #6 | (("over 50*" or "over 55*" or "over 60*" or "over 65*" or "over 70*" or "over 75*" or "over 80*" or "over 85*" or "over 90*" or "over 95*" or "over 100*")):ti,ab,kw | 10245 |
| #7 | ((quinquagenarian* or sexagenarian* or septuagenarian* or octogenarian* or nonagenarian* or centenarian* or supercentenarian* or senium)):ti,ab,kw | 200 |
| #8 | (Menopause):ti,ab,kw | 7040 |
| #9 | #1 or #2 or #3 or #4 or #5 or #6 or #7 or #8 | 609869 |
| #10 | MeSH descriptor: [Dancing] this term only | 206 |
| #11 | MeSH descriptor: [Dance Therapy] this term only | 97 |
| #12 | (dance or dancing):ti,ab,kw | 1316 |
| #13 | ((Dance NEAR/3 (exercise* or movement or therapy))):ti,ab,kw | 394 |
| #14 | #10 or #11 or #12 or #13 | 1316 |
| #15 | MeSH descriptor: [Accident Prevention] this term only | 135 |
| #16 | MeSH descriptor: [Accidental Falls] this term only | 1671 |
| #17 | ((fall or falls or falling or faller)):ti,ab,kw | 23307 |
| #18 | ((accident* NEAR/3 (fall* or prevention))):ti,ab,kw | 3808 |
| #19 | (fall* NEAR/3 (reduc* or risk)):ti,ab,kw | 4243 |
| #20 | #15 or #16 or #17 or #18 or #19 | 24976 |
| #21 | #9 and #14 and #20 in Cochrane Reviews, Trials | 112 |

**CINAHL**

| **#** | **Query** | **Results** |
| --- | --- | --- |
| S21 | S9 AND S14 AND S20 | 115 |
| S20 | S15 OR S16 OR S17 OR S18 OR S19 | 74,395 |
| S19 | TI ((fall* N3 reduc*) or (fall* N3 risk)) or AB ((fall* N3 reduc*) or (fall* N3 risk)) | 11,715 |
| S18 | TI ((accident* N3 fall*) or (accident* N3 prevention)) or AB ((accident* N3 fall*) or (accident* N3 prevention)) | 1,716 |
| S17 | TI (fall*) or AB (fall*) | 66,185 |
| S16 | MH accidental falls | 25,899 |
| S15 | MH accident prevention | 1,866 |
| S14 | S10 OR S11 OR S12 OR S13 | 6,793 |
| S13 | TI ((dance N3 exercise*) or (dance N3 movement) or (dance N3 therapy)) OR AB ((dance N3 exercise*) or (dance N3 movement) or (dance N3 therapy)) | 862 |
| S12 | TI (dance or dancing) OR AB (dance or dancing) | 5,090 |
| S11 | MH dance therapy | 983 |
| S10 | MH dancing | 3,572 |
| S9 | S1 OR S2 OR S3 OR S4 OR S5 OR S6 OR S7 OR S8 | 1,224,850 |
| S8 | TI (quinquagenarian* OR sexagenarian* OR septuagenarian* OR octogenarian* OR nonagenarian* OR centenarian* OR supercentenarian* OR senium) OR AB (quinquagenarian* OR sexagenarian* OR septuagenarian* OR octogenarian* OR nonagenarian* OR centenarian* OR supercentenarian* OR senium) | 2,556 |
| S7 | TI ("post-menopaus*" OR postmenopaus* OR menopaus*) OR AB ("post-menopaus*" OR postmenopaus* OR menopaus*) | 31,502 |
| S6 | MH menopause | 9,823 |
| S5 | TI ("over 50*" OR "over 55*" OR "over 60*" OR "over 65*" OR "over 70*" OR over 75*" OR "over 80*" OR "over 85*" OR "over 90*" OR "over 95*" OR "over 100*") OR AB ("over 50*" OR "over 55*" OR "over 60*" OR "over 65*" OR "over 70*" OR over 75*" OR "over 80*" OR "over 85*" OR "over 90*" OR "over 95*" OR "over 100*") | 1,432 |
| S4 | TI (older AND (person* OR people OR woman OR women OR female* OR man OR men OR male* OR adult* OR patient* OR population* OR subject*)) OR AB (older AND (person* OR people OR woman OR women OR female* OR man OR men OR male* OR adult* OR patient* OR population* OR subject*)) | 221,800 |
| S3 | TI (elder* OR eldest OR “old* age*” OR senior* OR aged OR geriatri*) OR AB (elder* OR eldest OR “old* age*” OR senior* OR aged OR geriatri*) | 390,088 |
| S2 | (MH middle aged+") | 5,609 |
| S1 | (MH "Aged+") | 931,950 |

**PEDro**

Abstract & Title: "older people" danc* fall*

Results = 12 records

**Supplementary File B: Inclusion and exclusion criteria**

|  | **Inclusion criteria** | **Exclusion criteria** |
| --- | --- | --- |
| **Population** | Adults 50 years and over, either identified as healthy, at risk of falls, or with co-morbidities. |  |
| **Intervention** | Peer-reviewed publications following an experimental, quasi-experimental, or non-experimental Interventions based on any type of dance, particularly in the experimental group Cost effectiveness of dance-based interventions Studies that are published in English language | Studies that do not have an intervention Studies that are qualitative Study protocols without published results, including conference abstracts |
| **Comparator** | Dance interventions where the experimental group is compared with a control group based on usual care, no intervention, or other non-dance fall prevention interventions (e.g. structured exercise programmes) Multicomponent intervention/s that have dance as a distinctive intervention in the intervention arm vs other group/s Studies without control group as long as outcomes were measured at baseline and post intervention | Multimodal studies where the study design does not enable to separate the effect of the dance component within the intervention, i.e., dance component not in the experimental group |
| **Outcomes** | Primary outcome: falls (rate of falls and number of falls)  Secondary outcomes: functional measures on balance (e.g. Berg Balance Score, One Leg Stance, Functional Reach, Timed Up and Go) and strength (e.g. Sit-to-Stand), generic health-related quality of life (e.g. European Quality of Life Instrument (EuroQoL EQ-5D)), and/or cost effectiveness evaluations |  |

**Supplementary File C: Sensitivity analyses using fixed-effect model to explore small-study effects**

**
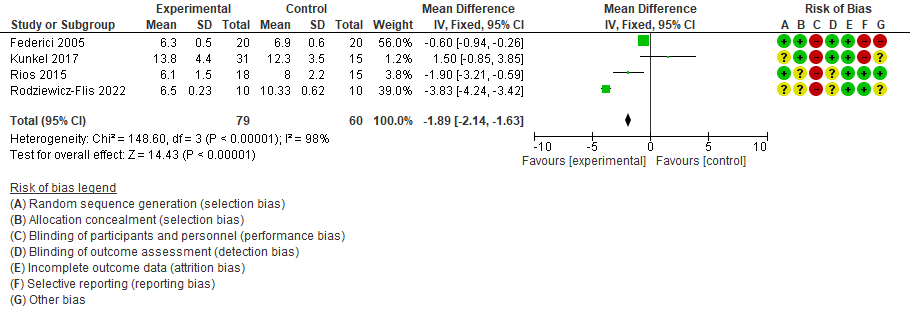
**

**Pooled mean differences, Timed Up and Go Dance versus usual care, 4 RCTs**

**
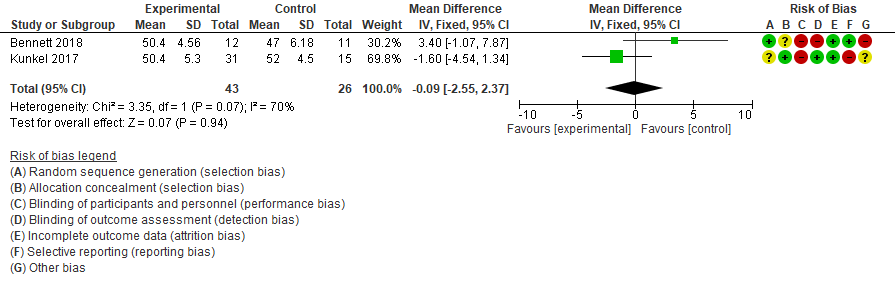
**

**Pooled mean differences, Berg Balance Scale Dance versus usual care, 2 RCTs**

**
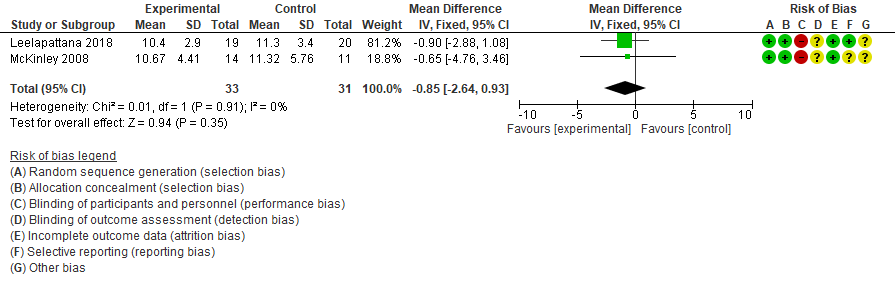
**

**Pooled mean differences, Sit to stand Dance versus exercise, 2 RCTs**

**
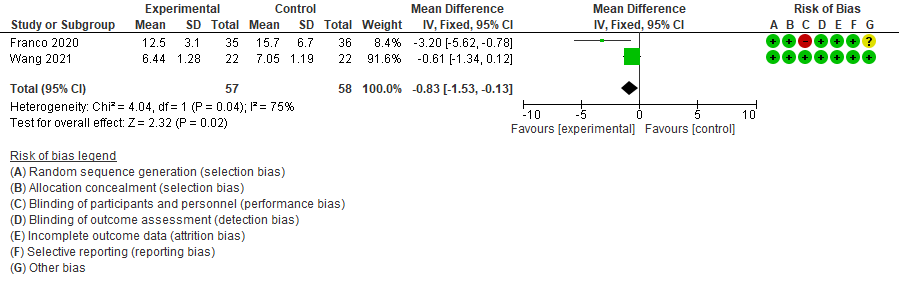
**

**Pooled mean differences, Sit to stand Dance versus education, 2 RCTs**

**Supplementary File D: Full summary characteristics of all included studies**

| Author, year | Study design (country) | Population N, condition, mean age (SD) | Type of dance | Intervention (setting), components and comparator | Provider of intervention | Duration of the programme |
| --- | --- | --- | --- | --- | --- | --- |
| Areeudomwong 2019 | RCT (Thailand) | 78 sedentary adults. CG: 67.33 (4.04), IG: 66.3 (4.33) | Cultural dance | Thai boxing dance programme (primary health): Programme consisted of warm-up, upper and lower limb stretching, and Thai dance boxing where participants stepped in a square-like direction following a Muay Thai song.  Comparator: Education (Falls Prevention booklet). | Trained instructor with four assistants | 50 minutes, 3x a week, for 4 weeks |
| Bennett 2018 | RCT (US) | 23 sedentary, non-disabled community-dwelling adults. 73.4 (8.4) | Low impact | Line dancing (community centre): Low impact dance with simple dance routines from novice line dance classes.  Comparator: Usual care. | Dance instructor | 1 hour, 2x a week, for 8 weeks |
| Britten 2017 | Mixed Methods (UK) | 38 healthy, community dwelling adults. 77.3 (8.4) | Low impact | Contemporary dance (local community facilities): A sequence of movements to encourage tactile interaction and improvisation. Participants showcase the dance to neighbourhood at end of programme. It also involved sharing of experiences and discussions around the barriers that older adults may find during physical exercise.  Comparator: No comparator. | Dance instructor | 90 minutes, 1x a week, for 8 weeks |
| Buransri 2021 | Quasi-experimental (Thailand) | 90 healthy, community dwelling adults. CG: 64.20 (4.5), IG: 63.64 (4.6) | Cultural dance | Traditional Srichiangmai dance (School for older people): 15 Traditional Srichiangmai dancing postures techniques with static stretching.  Comparator: Exercise (Walking). | Sports scientists | 30 minutes, 3x a week, for 12 weeks |
| Charras 2020 | Quasi-experimental (France) | 23 older adults w/ Dementia. 83.47 (5.40) | Dance exercise | Dance exercise (Day care centre for people with dementia): Sensory and muscular awakening, coordination exercises and improvisations, dance exercises (tango, waltz, and classical dance movements).  Comparator: Usual care. | Dance instructor with a nursing background, and 2 facilitators | 50 minutes, 1x a week, for 24 weeks |
| da Silva Borges 2014 | RCT (Brazil) | 59 sedentary adults in long-stay institutions. CG: 67 (7.7), IG: 68 (8.3) | Ballroom and Latin American dance | Ballroom dancing programme (Long-stay institution): A range of higher intensity rhythm, with relaxation period accompanied by music.  Comparator: Usual care. | NR | 50 minutes, 3x a week, for 12 weeks |
| de Natale 2017 | Quasi-experimental (Italy) | 16 adults w/ Parkinson's disease. 67 ± 6.9 CG: 70 (3.16), IG: 66 (9.15) | Ballroom and Latin American dance | Tango (Dance hall): new Tango steps of increasing difficulties and free partnered dance.  Comparator: Exercise (Traditional rehabilitation). | NR | 60 minutes, 2x a week, for 10 weeks |
| Federici 2005 | RCT (Italy) | 40 healthy, community dwelling adults. CG: 63.5 (3.7), IG: 62.7 (4.1) | Dance exercise | Dance exercise (Recreation centre): General conditioning activities (light exercises, stretching and breathing exercises), followed by specific exercises for static and dynamic balance.  Comparator: Usual care. | NR | 1 hour, 2x a week, for 3 months |
| Filar-Mierzwa 2016 | Observational (Poland) | 24 healthy, sedentary living adults. 66.4 | Dance exercise | Dance exercise (Senior living facility): Dancing-gymnastic exercises, included basic steps and figures of a folk dance, ballroom dance, integration dance, and dances of foreign nations.  Comparator: No comparator | Trained instructors | 45 minutes, 1x a week, for 3 months |
| Filar-Mierzwa 2021 | Quasi-experimental (Poland) | 39 healthy, sedentary living adults. CG: 67, IG: 67.45 | Dance exercise | Dance exercise (Senior living facility): Dancing-gymnastic exercises, included basic steps and figures of a folk dance, ballroom dance, integration dance, and dances of foreign nations.  Comparator: Exercise (general). | Trained instructors | 45 minutes, 1x a week, for 3 months |
| Franco 2020 | RCT (Brazil) | 82 healthy, community dwelling adults. 69 (6.6) CG: 70 (6.2), IG: 68.6 (7.2) | Cultural dance | Senior Dance (DanSE): A range of choreographies, including rhythmic and simple movements with rhythmic folk songs.  Comparator: Education. | Dance instructor | 1 hour, 2x a week, for 12 weeks |
| Goldsmith 2021 | Cost effectiveness analysis (UK) | 1194 older adults. 77 | Dance exercise | Dance to Health(local community facilities): Exercises from both Otago and FaME, as these programmes allow for progression and flexibility.  Comparator: No comparator. | Dance instructor | 2.5 hours, 1x a week, for 56 weeks |
| Hackney 2013 | Observational (US) | 13 adults with vision impairment. 86.9 (5.9) | Ballroom and Latin American dance | Adapted tango (Senior living facility): Classes in tango with upbeat music and standing warm-ups. Rather than using a traditional ballroom frame, participants were taught to lead and follow steps while holding the elbows facing one another, maintaining their forearms parallel to the floor.  Comparator: No control group. | Dance instructor, personal trainer | 1.5 hours, 1-2x a week, for 12 weeks |
| Hackney 2015 | Quasi-experimental (US) | 32 adults with vision impairment. 79.3 (11) Tango: 84.9 (9), FallProof: 74.8 (11.2) | Ballroom and Latin American dance | Adapted tango (Senior living facility): Classes in tango with upbeat music and standing warm-ups. Rather than using a traditional ballroom frame, participants were taught to lead and follow steps while holding the elbows facing one another, maintaining their forearms parallel to the floor.  Comparator: Exercise (FallProof classes). | Dance instructor, personal trainer | 1.5 hours, 1-2x a week, for 12 weeks |
| Hamacher 2016 | RCT (Germany) | 32 healthy adults. CG: 68.33 (3.17), IG: 66.73 (3.33) | Ballroom and Latin American dance | Dance programme: Line Dance, Jazz Dance, Rock 'n' Roll, Latin-American Dance and Square Dance.  Comparator: Exercise (Strength endurance and flexibility training). | NR | 90 minutes, 2x a week, for 6 months |
| Hofgaard 2019 | RCT (UK) | 27 healthy adults. CG: 74 (4), IG: 75 (5) | Cultural dance | Faroese chain dance programme (Indoor recreation centre): A medieval ring dance organised as a chain where all dancers are positioned side-by-side holding hands firmly with the dancer to the left and right forming a ring or circle.  Comparator: Usual care. | NR | 30-45 minutes for 6 weeks |
| Kaewjoho 2020 | Observational (Thailand) | 61 community dwelling adults. 72.9 (5.7) | Cultural dance | Thai dance exercise: The Thai dance exercises were performed with a video demonstration of the standard traditional Thai dance using eight songs.  No comparator. | NR | 50 minutes, 3x a week, for 6 weeks |
| Kalyani 2020 | Quasi-experimental (New Zealand) | 33 adults with Parkinson's Disease. CG: 66.5 (7.7), IG: 65.24 (11.88) | Dance exercise | Dance for Parkinson’s Disease® (DfPD®) programme (University): Standing dance with support and movement across the floor. Participants could attend with a family member, friend, or caregiver.  Comparator: Usual care. | DfPD-trained instructors | 1 hour, 2x a week, for 12 weeks |
| Krampe 2010 | Observational (US) | 11 healthy adults (Mean age: NR) | Dance therapy | The Lebed Method Dance therapy (Senior living facility): Low-impact dance with upbeat participant-specific music.  Comparator: No comparator. | Trained instructors | 45 minutes, 3x a week, for 6 weeks |
| Kunkel 2017 | RCT (UK) | 51 adults with Parkinson's disease. CG: 69.7 (6), IG: 71.3 (7.7) | Ballroom and Latin American dance | Mixed dances programme (Local dance centre): Ballroom (social foxtrot, waltz and tango) and Latin American (cha cha, rock-and-roll and rumba).  Comparator: Usual care. | Professional dancer | 1 hour, 2x a week, for 10 weeks |
| Leelapattana 2018 | RCT (Thailand) | 39 self-ambulatory women. CG: 66.9 (5.6), IG: 66.4 (4.2) | Cultural dance | Thai classical dance exercises (Hospital): Thai classical music following multimedia instructions.  Comparator: Exercise (Arm-swing exercise). | NR | 10 minutes for 12 weeks |
| Li 2022 | RCT (South Korea) | 40 healthy adults. CG: 61.75 (1.11), IG: 61.8 (1.23) | Ballroom and Latin American dance | Cha-cha dance training (University): The training content mainly consisted of basic steps of performing repetitive symmetrical movements.  Comparator: Usual care. | NR | 90 minutes, 3x a week, for 12 weeks |
| Machacova 2017 | RCT (Czech Republic) | 189 nursing home residents. CG: 82.88 (8.16), IG: 83.03 (9.10) | Ballroom and Latin American dance | EXDASE (EXercise DAnce for Seniors) (Nursing home): Basic steps and combinations of ballroom dances, including foxtrot, waltz, cha-cha-cha, cancan, etc.  Comparator: Usual care. | Dance instructor | 1 hour, 1x a week, for 3 months |
| McKee 2013 | Quasi-experimental (US) | 33 adults with Idiopathic definite Parkinson's Disease. CG: 74.4 (6.5), IG: 68.4 (7.5) | Ballroom and Latin American dance | Tango (Senior independent living communities): Rhythmic enhancement exercises with novel step elements.  Comparator: Education. | Dance instructor | 90 minutes |
| McKinley 2008 | RCT (Canada) | 25 healthy, living independently adults. CG: 74.6 (8.4), IG: 78.07 (7.6) | Ballroom and Latin American dance | Argentine Tango dance programme (Senior centre): Individual exercises that focused on the components of the basic tango.  Comparator: Exercise (Walking). | Dance instructor | 2 hours, 2x a week, for 10 weeks |
| Merom 2016 | RCT (Australia) | 530 community dwelling adults. Age >80: 208 (39%) | Ballroom and Latin American dance | Social dance (Self-care retirement villages, clusters): Participants in the 12 intervention villages were offered one of two major social dancing styles: Folk dancing (five villages), which included dances from the UK, US France, Italy, Israel, and Greece; or ballroom dancing (seven villages), which included dances such as Rock and Roll, Foxtrot, Waltz, Salsa, and Rumba.  Comparator: Usual care. | Dance instructor | 1 hour, 2x a week, for 12 months |
| Noopud 2019 | RCT (Thailand) | 43 community dwelling women. CG: 68.29 (5.82), IG: 67.5 (5.39) | Cultural dance | Thai traditional dance: Basic movements of the upper and lower extremities, weight shifting skills, slow steps, and coordination of movement with Thai traditional music playing in the background.  Comparator: Usual care. | NR | 30-60 minutes for 12 weeks |
| Nur 2022 | RCT (Indonesia) | 41 community dwelling adults. CG: 71.6 (10.11), IG: 67.81 (7.731) | Cultural dance | Molong Kopi (Senior long-term care centre): A traditional dance from Bondowoso city of Indonesia about gratitude for the agricultural products by coffee farmers. Manual book was written based on the original movements of the dance and also a video tutorial on performing the dance.  Comparator: Usual care. | Research assistants | 15 minutes for 8 weeks |
| O'Toole 2015 | Mixed methods (Ireland) | 62 community dwelling adults. Aged over 70 (n = 25; 41.7% | Low impact | Contemporary dance (Local dance theatre and community centre): Routines are accompanied by a wide variety of music including jazz, classical and contemporary pieces. Movements incorporate use of the arms, torso, core and legs such as stretching, leg lifts, stepping forward, backward and side-stepping, lunges and heel rises.  Comparator: No comparator. | Dance instructors | 1x a week for 6 weeks |
| Pope 2019 | Quasi-experimental (US) | 163 community dwelling older adults. CG : 70.7( 6.9); IG: 73.4 (7.7) | Dance therapy | The Lebed Method Dance therapy: A dance-based therapy that employs low-impact dance and was developed for persons with lymphedema and physical limitations. Lymphatic system warm up (breathing exercises, full body active range of motion exercises, and stretches) followed by dance sequences of low-impact ballet, jazz, and aerobic movements.  Comparator: Exercise (Stay Active and Independent for Life (SAIL)). | Trained instructors | 1 hour, 2-3 a week, for 8-10 weeks |
| Rawson 2019 | RCT (US) | 96 adults with Idiopathic Parkinson's disease. 78.97 (20.67) | Ballroom and Latin American dance | Argentine tango (University): An adapted Argentine Tango curriculum for PD. Dance partners were spouses, caregivers, volunteers, and laboratory staff.  Comparator: Exercise (Stretching and treadmill). | Physical therapist, Trained instructors, laboratory staff | 1 hour, 2x a week, for 12 weeks |
| Rios 2015 | RCT (Canada) | 33 adults with Idiopathic Parkinson's disease. CG: 64.3 (8.1), IG: 63.2 (9.9) | Ballroom and Latin American dance | Argentine Tango (University): Standard footwork exercises. All participants learned a 90 s dance routine then performed it for friends and family.  Comparator: Usual care. | Dance instructor | 1 hour, 2x a week, for 12 weeks |
| Rodgrigues-Krause 2018 | RCT (Brazil) | 30 sedentary women. 65(5). CG (Stretch): 66 (61-70), Dance: 66 (63-70), Walk: 64 (62-65) | Dance exercise | Structured dancing: Functional and fitness elements (balance, flexibility, muscle power, aerobic conditioning, etc.).  Comparator: Exercise (Walking and stretching). | Specialised instructors | 1 hour, 1-3x a week for 8 weeks |
| Rodziewicz-Flis 2022 | RCT (Poland) | 30 community-dwelling women. 73.3 (4.5), CG: 73.4 (5.0), Dance: 72.1 (4.1), Balance: 74.3 (4.6) | Dance exercise | Dance and balance training (Community centre): Choreography learning, dance figures, and fitness exercises. The training ended with static stretching and relaxation exercises.  Comparator: Exercise and usual care. | Dance instructor, physiotherapist | 50 minutes, 3x a week, for 12 weeks |
| Shigematsu 2002 | Quasi-experimental (Japan) | 38 community dwelling, healthy independent women. CG: 79.8 (5.0), IG: 78.6 (4.0) | Dance exercise | Dance-based aerobics (Community centre): Dance-based aerobic exercises with cool-down activities and accompanied by music.  Comparator: Usual care. | Exercise specialist | 1 hour, 3x a week, for 3 months |
| Sohn 2018 | Observational (South Korea) | 15 older adults. 72 (5.4) | Ballroom and Latin American dance | Dancesport (Senior welfare centre): Rumba, Jive, and Cha-cha-cha.  No comparator. | NR | 50 minutes, 3x a week, for 15 weeks |
| Tillmann 2020 | Quasi-experimental (Brazil) | 20 adults with Parkinson's disease. 66.4 (10.7) | Ballroom and Latin American dance | Brazilian samba (Rehabilitation centre): Lessons were conducted in a large room that was considered appropriate for patients with Parkinson’s Disease, a floor without deformities and chairs to allow participants to rest. Music was set at a moderate volume.  Comparator: Usual care. | Dance instructor | 1 hour, 2x a week, for 12 weeks |
| Vella-Burrows 2021 | Mixed Methods (UK) | 67 older adults | Dance exercise | Dance to Health (local community facilities) : A falls prevention programme that integrates the remote but connected professional communities of physiotherapy and artistic dance. The theoretical and practice based principles are taken from the FaME and Otago falls-prevention exercise programmes and embedded into creative dance programmes.  No comparator. | Dance artists | 90 minutes, 2-3x a week, for 6 months |
| Ventura 2016 | Quasi-experimental (US) | 15 adults with Parkinson's disease. CG: 71.8 (3.6), IG: 70.4 (5.5) | Dance exercise | Dance for Parkinson’s Disease® (DfPD®) programme: A series of choreographed dance movements (dance forms included ballet, jazz, Broadway style dance), improvisational movement such as mirroring, or movement across the floor.  Comparator: Usual care. | DfPD-trained instructors | 1.25 hours, 1x a week, for 5 months |
| Wang 2021 | RCT (China) | 44 community dwelling, healthy independent adults. 64.1 (4.02) | Low impact | Modified tap dance program (MTD): MTD practice and cooling down.  Comparator: Education. | Dance instructor | 1 hour, 3x a week, for 12 weeks |
| Weighart 2020 | Quasi-experimental (US) | 17 community dwelling, healthy independent adults. CG: 65.9 (11.9), IG: 73.3 (10.6) | Low impact | Ballet (Dance theatre): Traditional classical ballet technique. Movement ‘combinations’ alongside verbal cues from the lead teacher.  Comparator: Usual care. | Dance instructor | 1 hour, 2x a week, for 10 weeks |

* NR: Not reported; RCT: Randomised controlled trial; UK: United Kingdom; US: United States

**Supplementary File E: Risk of Bias assessments on included studies**

**Risk of bias assessment for randomised studies, Cochrane Risk of Bias (RoB) tool (N=19)**

**
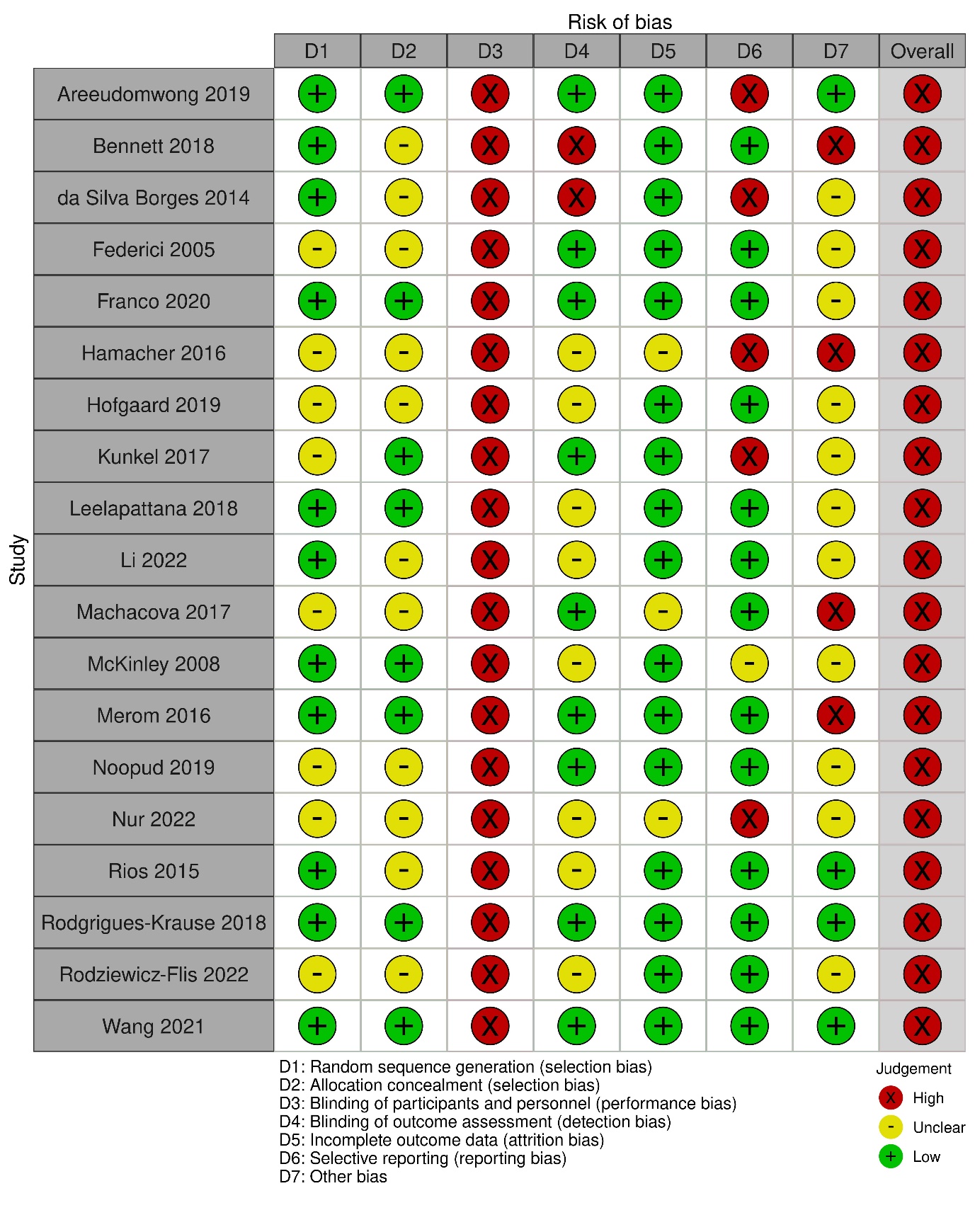
**

**Risk of bias assessment for non-randomised studies,** **Risk Of Bias In Non-randomized Studies – of Interventions (ROBINS-I) tool (N=13)**

**
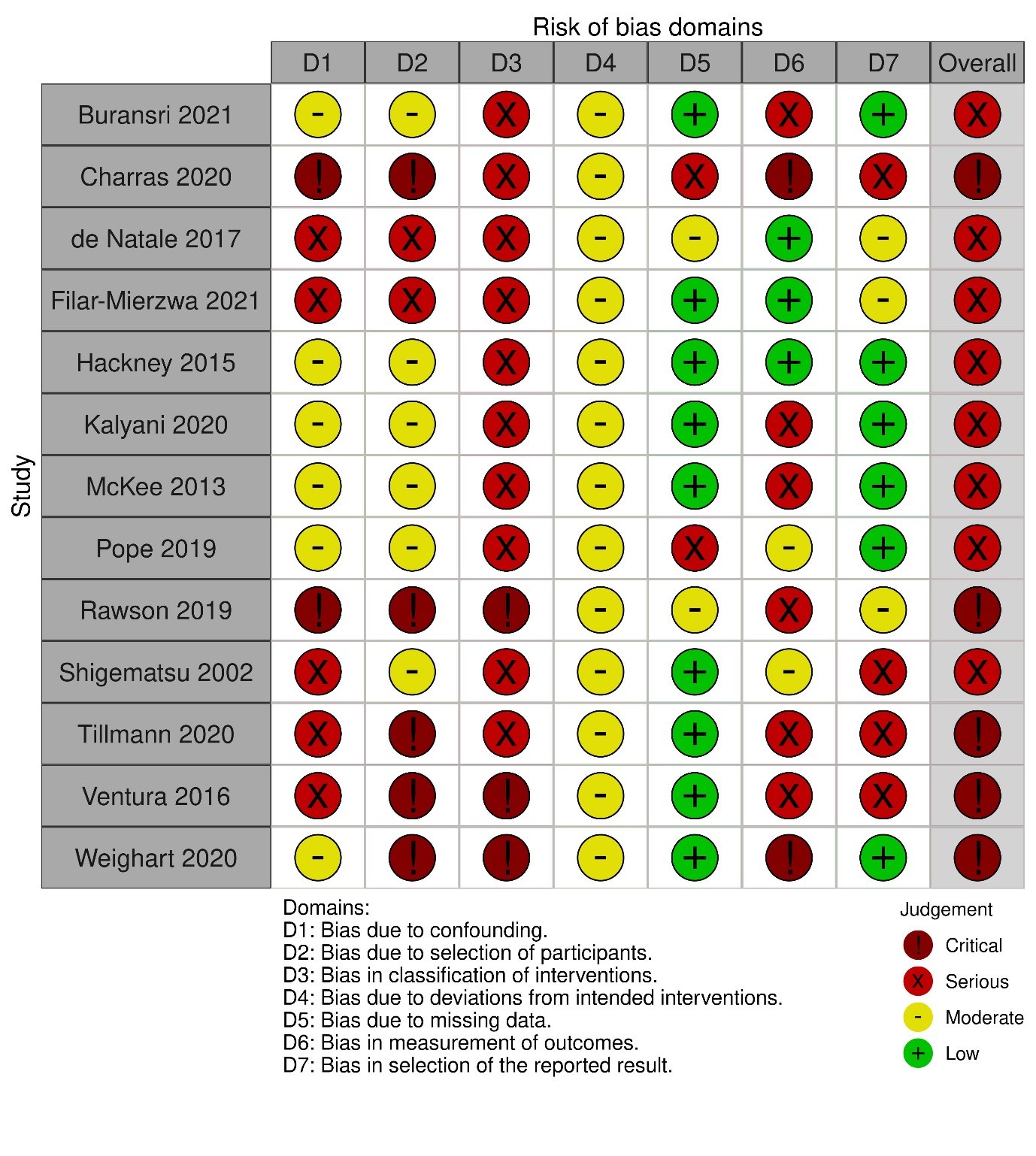
**

**Risk of bias assessment for observational studies, Risk Of Bias In Non-randomized Studies – of Exposure (ROBINS-E) (N=6)**

**
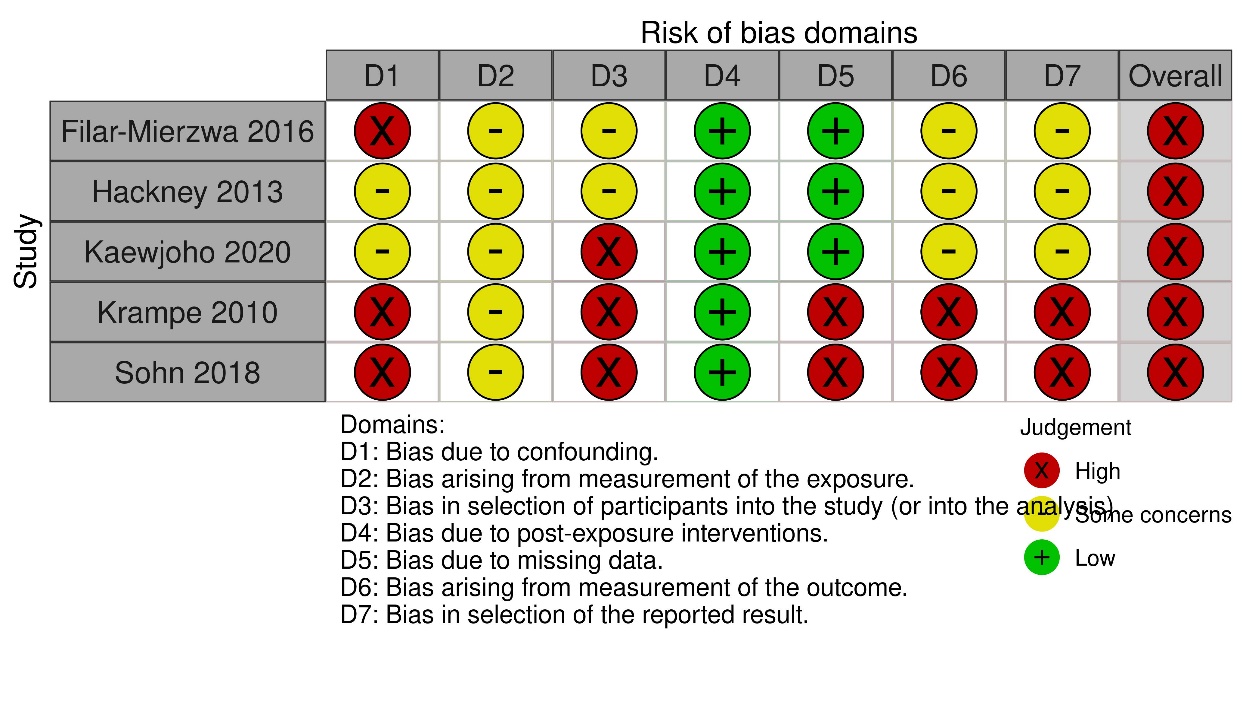
**

**Risk of bias assessment for mixed methods studies, Mixed Methods Appraisal Tool (MMAT) (N=3)**

|  | Britten 2017 | O’Toole 2015 | Vella Burrows 2021 |
| --- | --- | --- | --- |
| S1. Are there clear research questions? | Yes | Yes | Yes |
| S2. Do the collected data allow to address the research questions? | Yes | Yes | Yes |
| 1. Qualitative  1.1. Is the qualitative approach appropriate to answer the research question? | Yes | Yes | Yes |
| 1.2. Are the qualitative data collection methods adequate to address the research question? | Yes | Yes | Yes |
| 1.3. Are the findings adequately derived from the data? | Yes | Yes | Yes |
| 1.4. Is the interpretation of results sufficiently substantiated by data? | Yes | Yes | Yes |
| 1.5. Is there coherence between qualitative data sources, collection, analysis and interpretation? | Yes | Yes | Yes |
| 2. Quantitative randomized controlled trials  2.1. Is randomization appropriately performed? | NA | NA | NA |
| 2.2. Are the groups comparable at baseline? | NA | NA | NA |
| 2.3. Are there complete outcome data? | NA | NA | NA |
| 2.4. Are outcome assessors blinded to the intervention provided? | NA | NA | NA |
| 2.5 Did the participants adhere to the assigned intervention? | NA | NA | NA |
| 3. Quantitative nonrandomized  3.1. Are the participants representative of the target population? | No | No | No |
| 3.2. Are measurements appropriate regarding both the outcome and intervention (or exposure)? | Yes | No | Yes |
| 3.3. Are there complete outcome data? | Yes | No | No |
| 3.4. Are the confounders accounted for in the design and analysis? | No | No | No |
| 3.5. During the study period, is the intervention administered (or exposure occurred) as intended? | Yes | Yes | Yes |
| 4. Quantitative descriptive  4.1. Is the sampling strategy relevant to address the research question? | NA | NA | NA |
| 4.2. Is the sample representative of the target population? | NA | NA | NA |
| 4.3. Are the measurements appropriate? | NA | NA | NA |
| 4.4. Is the risk of nonresponse bias low? | NA | NA | NA |
| 4.5. Is the statistical analysis appropriate to answer the research question? | NA | NA | NA |
| 5. Mixed methods  5.1. Is there an adequate rationale for using a mixed methods design to address the research question? | No | Yes | Yes |
| 5.2. Are the different components of the study effectively integrated to answer the research question? | Yes | Yes | No |
| 5.3. Are the outputs of the integration of qualitative and quantitative components adequately interpreted? | Yes | Yes | No |
| 5.4. Are divergences and inconsistencies between quantitative and qualitative results adequately addressed? | Yes | Yes | Yes |
| 5.5. Do the different components of the study adhere to the quality criteria of each tradition of the methods involved? | No | No | No |

**Risk of bias assessment for cost-effectiveness analysis study, Drummond checklist (N=1)**

|  | Goldsmith 2021 |
| --- | --- |
| 1. Was the research question stated? | Yes |
| 2. Was the economic importance of the research question stated? | Yes |
| 3. Was/were the viewpoint(s) of the analysis clearly stated and justified? | No |
| 4. Was a rationale reported for the choice of the alternative programmes or interventions compared? | No |
| 5. Were the alternatives being compared clearly described? | No |
| 6. Was the form of economic evaluation stated? | Yes |
| 7. Was the choice of form of economic evaluation justified in relation to the questions addressed? | Yes |
| 8. Was/were the source(s) of effectiveness estimates used stated? | Yes |
| 9. Were details of the design and results of the effectiveness study given (if based on a single study)? | No |
| 10. Were details of the methods of synthesis or meta-analysis of estimates given (if based on an overview of a number of effectiveness studies)? | No |
| 11. Were the primary outcome measure(s) for the economic evaluation clearly stated? | No |
| 12. Were the methods used to value health states and other benefits stated? | No |
| 13. Were the details of the subjects from whom valuations were obtained given? | No |
| 14. Were productivity changes (if included) reported separately? | NA |
| 15. Was the relevance of productivity changes to the study question discussed? | NA |
| 16. Were quantities of resources reported separately from their unit cost? | No |
| 17. Were the methods for the estimation of quantities and unit costs described? | No |
| 18. Were currency and price data recorded? | No |
| 19. Were details of price adjustments for inflation or currency conversion given? | No |
| 20. Were details of any model used given? | No |
| 21. Was there a justification for the choice of model used and the key parameters on which it was based? | No |
| 22. Was the time horizon of cost and benefits stated? | No |
| 23. Was the discount rate stated? | No |
| 24. Was the choice of rate justified? | No |
| 25. Was an explanation given if cost or benefits were not discounted? | No |
| 26. Were the details of statistical test(s) and confidence intervals given for stochastic data? | No |
| 27. Was the approach to sensitivity analysis described? | No |
| 28. Was the choice of variables for sensitivity analysis justified? | No |
| 29. Were the ranges over which the parameters were varied stated? | No |
| 30. Were relevant alternatives compared? (That is, were appropriate comparisons made when conducting the incremental analysis?) | No |
| 31. Was an incremental analysis reported? | No |
| 32. Were major outcomes presented in a disaggregated as well as aggregated form? | No |
| 33. Was the answer to the study question given? | Yes |
| 34. Did conclusions follow from the data reported? | No |
| 35. Were conclusions accompanied by the appropriate caveats? | No |
| 36. Were generalisability issues addressed? | Yes |

**Supplementary File F: Summary of outcomes on included studies, effect sizes where available**

| Author, year | Study design | Comparison | Assessment time point | Outcome | Measure | Unit | Summary data, where available | | | | | | Calculated MD | | | | Vote counting | |
| --- | --- | --- | --- | --- | --- | --- | --- | --- | --- | --- | --- | --- | --- | --- | --- | --- | --- | --- |
|  |  |  |  |  |  |  | **IG: Mean** | **IG: SD** | **IG: No. of participants** | **CG: Mean** | **CG: SD** | **CG: No. of participants** | **MD** | **95% CI** | **P value** |  | |  |
| Areeudomwong 2019 | RCT | Education | 16 weeks | Functional | Timed up and go (TUG) | Seconds | NR | NR | 39 | NR | NR | 39 | NR | NR | NR | 1 | |  |
|  |  |  |  |  | Romberg test | Seconds | NR | NR | 39 | NR | NR | 39 | NR | NR | NR | 0 | |  |
|  |  |  |  | Strength | Left hip flexors | Kg | 6.07 | 0.81 | 39 | 5.6 | 0.79 | 39 | 0.47 | [0.11, 0.83] | P = 0.009 | 1 | |  |
|  |  |  |  |  | Right hip flexors | Kg | 6.37 | 1.94 | 39 | 5.47 | 1.47 | 39 | 0.9 | [0.14, 1.66] | P = 0.02 | 1 | |  |
|  |  |  |  |  | Left hip extensors | Kg | 5.95 | 0.86 | 39 | 5.46 | 0.84 | 39 | 0.49 | [0.11, 0.87] | P = 0.01 | 1 | |  |
|  |  |  |  |  | Right hip extensors | Kg | 6.09 | 0.74 | 39 | 5.62 | 1.16 | 39 | 0.47 | [0.04, 0.90] | P = 0.03 | 1 | |  |
|  |  |  |  |  | Left knee flexors | Kg | 5.62 | 1.45 | 39 | 4.77 | 0.85 | 39 | 0.85 | [0.32, 1.38] | P = 0.002 | 1 | |  |
|  |  |  |  |  | Right knee extensors | Kg | 5.56 | 1.58 | 39 | 4.67 | 1.05 | 39 | 0.89 | [0.29, 1.49] | P = 0.003 | 1 | |  |
|  |  |  |  |  | Left knee extensors | Kg | 5.24 | 1.11 | 39 | 4.62 | 0.98 | 39 | 0.62 | [0.16, 1.08] | P = 0.009 | 1 | |  |
|  |  |  |  |  | Right knee flexors | Kg | 5.37 | 1.2 | 39 | 4.56 | 1.07 | 39 | 0.81 | [0.31, 1.31] | P = 0.002 | 1 | |  |
|  |  |  |  |  | Left ankle dorsiflexors | Kg | 4.1 | 0.66 | 39 | 3.64 | 0.34 | 39 | 0.46 | [0.23, 0.69] | P = 0.0001 | 1 | |  |
|  |  |  |  |  | Right ankle dorsiflexors | Kg | 4.01 | 0.58 | 39 | 3.71 | 0.31 | 39 | 0.3 | [0.09, 0.51] | P = 0.004 | 1 | |  |
|  |  |  |  |  | Left ankle pantarflexors | Kg | 4.07 | 0.59 | 39 | 3.71 | 0.38 | 39 | 0.36 | [0.14, 0.58] | P = 0.001 | 1 | |  |
|  |  |  |  |  | Right ankle pantarflexors | Kg | 4.06 | 0.61 | 39 | 3.65 | 0.34 | 39 | 0.41 | [0.19, 0.63] | P = 0.0002 | 1 | |  |
| Bennett 2018 | RCT | Usual care | 8 weeks | Functional | Berg Balance Scale (BBS) | Points | 50.4 | 4.56 | 12 | 47 | 6.18 | 11 | 3.4 | [-1.07, 7.87] | P = 0.14 | 1 | |  |
|  |  |  |  |  | Short Physical Performance Battery (SPPB) | Points | 9.17 | 2.32 | 12 | 7.18 | 2.08 | 11 | 1.99 | [0.19, 3.79] | P = 0.03 | 1 | |  |
|  |  |  |  | Strength | Knee extensor strength | Kg | 0.14 | 0.04 | 12 | 0.09 | 0.03 | 11 | 0.05 | [0.02, 0.08] | P = 0.0007 | 1 | |  |
|  |  |  |  |  | Knee flexor strength | Kg | 0.11 | 0.06 | 12 | 0.08 | 0.03 | 11 | 0.03 | [-0.01, 0.07] | P = 0.12 | 1 | |  |
| Britten 2017 | Observational, pre-post | No control | 8 weeks | Concern about falling | Falls efficacy scale-international (FES-I) | Points | 23.7 | 8.6 | 20 | No control | No control | No control | No control | No control | No control | 1 | |  |
|  |  |  |  | Functional | Timed up and go (TUG) | Seconds | 7.7 | 2.8 | 17 | No control | No control | No control | No control | No control | No control | 1 | |  |
| Buransri 2021 | QE | Exercise | 12 weeks | Functional | Functional reach test | cm | 16.6 | 1.9 | 45 | 14.2 | 1.9 | 45 | 2.4 | [1.61, 3.19] | P < 0.00001 | 1 | |  |
|  |  |  |  |  | Timed up and go (TUG) | Seconds | 7.17 | 2.1 | 45 | 7.24 | 1.8 | 45 | -0.07 | [-0.88, 0.74] | P = 0.87 | 1 | |  |
|  |  |  |  |  | 6-minute walk test (6MWT) | Meters | 495.85 | 54.9 | 45 | 481 | 53 | 45 | 14.85 | [-7.45, 37.15] | P = 0.19 | 1 | |  |
|  |  |  |  |  | Chair stand test | Reps | 18.7 | 1.9 | 45 | 18.2 | 1.7 | 45 | 0.5 | [-0.24, 1.24] | P = 0.19 | 1 | |  |
|  |  |  |  | Strength | Arm curl test | Reps | 18.5 | 1.3 | 45 | 19.27 | 1.6 | 45 | -0.77 | [-1.37, -0.17] | P = 0.01 | 0 | |  |
| Charras 2020 | QE | Usual care | 12 weeks | Functional | Timed up and go (TUG) | Seconds | 20.49 | 8 | 21 | 18 | 4.96 | 21 | 2.49 | [-1.54, 6.52] | P = 0.23 | 0 | |  |
|  |  |  |  | Quality of life | QoL in Alzheimer's Disease (QoL-AD) | Points | 36.47 | 4.79 | 21 | 36.95 | 4.69 | 21 | -0.48 | [-3.35, 2.39] | P = 0.74 | 0 | |  |
| Da Silva Borges 2014 | RCT | Usual care | 12 weeks | Falls | No. of falls | Number | NR | NR | 30 | NR | NR | 29 | NR | NR | P < 0.00001 | 1 | |  |
|  |  |  |  | Functional | Corporal balance | Kg | 6.14 | 3.29 | 30 | 6.1 | 6.3 | 29 | 0.04 | [-2.54, 2.62] | P = 0.98 | 1 | |  |
| De Natale 2017 | QE | Exercise | 18 weeks | Functional | Berg Balance Scale (BBS) | Points | 51.5 | 5.07 | 9 | 48.5 | 7 | 7 | 3 | [-3.15, 9.15] | P = 0.34 | 1 | |  |
|  |  |  |  |  | Timed up and go (TUG) | Seconds | 10 | 2.35 | 9 | 12.57 | 4.05 | 7 | -2.57 | [-5.94, 0.80] | P = 0.14 | 1 | |  |
|  |  |  |  |  | 4-Square Step Test | Seconds | 11.35 | 2.12 | 9 | 11.89 | 2.95 | 7 | -0.54 | [-3.13, 2.05] | P = 0.68 | 0 | |  |
|  |  |  |  |  | 6-minute walk test (6MWT) | Meters | 377.25 | 124.94 | 9 | 324.33 | 89.1 | 7 | 52.92 | [-52.05, 157.89] | P = 0.32 | 1 | |  |
| Federici 2005 | RCT | Usual care | 12 weeks | Functional | Tinetti | Points | 23 | 3 | 20 | 21 | 2 | 20 | 2 | [0.42, 3.58] | P = 0.01 | 1 | |  |
|  |  |  |  |  | Romberg test | Seconds | 59.7 | 10.8 | 20 | 54.8 | 11.1 | 20 | 38.7 | [33.89, 43.51] | P < 0.00001 | 1 | |  |
|  |  |  |  |  | Improved Romberg test | Seconds | 7.2 | 1.6 | 20 | 6.4 | 1.7 | 20 | 0.8 | [-0.22, 1.82] | P = 0.13 | 1 | |  |
|  |  |  |  |  | Timed up and go (TUG) | Seconds | 6.3 | 0.5 | 20 | 6.9 | 0.6 | 20 | -0.6 | [-0.94, -0.26] | P = 0.0006 | 1 | |  |
| Filar-Mierzwa 2016 | Observational, pre-post | No control | 12 weeks | Functional | Postural stability | Points | 1.15 | 0.43 | 24 | No control | No control | No control | No control | No control | No control | 0 | |  |
|  |  |  |  |  | Limits of Stability (LOS) | degrees/second | 34.2 | 11.91 | 24 | No control | No control | No control | No control | No control | No control | 1 | |  |
|  |  |  |  |  | Fall Risk Test (FRT M–CTSIB) eyes open foam surface | degrees/second | 1.32 | 0.32 | 24 | No control | No control | No control | No control | No control | No control | 0 | |  |
|  |  |  |  |  | Fall Risk Test (FRT M–CTSIB) eyes open firm surface | degrees/second | 0.64 | 0.22 | 24 | No control | No control | No control | No control | No control | No control | 0 | |  |
|  |  |  |  |  | Fall Risk Test (FRT M–CTSIB) eyes closed foam surface | degrees/second | 2.45 | 0.59 | 24 | No control | No control | No control | No control | No control | No control | 0 | |  |
|  |  |  |  |  | Fall Risk Test (FRT M–CTSIB) eyes closed firm surface | degrees/second | 0.69 | 0.21 | 24 | No control | No control | No control | No control | No control | No control | 0 | |  |
| Filar-Mierzwa 2021 | QE | Exercise | 12 weeks | Functional | Postural stability | Points | 1.15 | 0.43 | 24 | 1.65 | 0.56 | 24 | -0.5 | [-0.78, -0.22] | P = 0.0005 | 0 | |  |
|  |  |  |  |  | Limits of Stability (LOS) | degrees/second | 34.2 | 11.91 | 24 | 36.42 | 12.09 | 24 | -2.22 | [-9.01, 4.57] | P = 0.52 | 0 | |  |
|  |  |  |  |  | Fall Risk Test (FRT M–CTSIB) eyes open foam surface | degrees/second | 1.32 | 0.32 | 24 | 1.37 | 0.22 | 24 | -0.05 | [-0.21, 0.11] | P = 0.53 | 0 | |  |
|  |  |  |  |  | Fall Risk Test (FRT M–CTSIB) eyes open firm surface | degrees/second | 0.64 | 0.22 | 24 | 0.8 | 0.32 | 24 | -0.16 | [-0.32, -0.00] | P = 0.04 | 0 | |  |
|  |  |  |  |  | Fall Risk Test (FRT M–CTSIB) eyes closed foam surface | degrees/second | 2.45 | 0.59 | 24 | 2.39 | 0.47 | 24 | 0.06 | [-0.24, 0.36] | P = 0.70 | 1 | |  |
|  |  |  |  |  | Fall Risk Test (FRT M–CTSIB) eyes closed firm surface | degrees/second | 0.69 | 0.21 | 24 | 0.95 | 0.33 | 24 | -0.26 | [-0.42, -0.10] | P = 0.001 | 0 | |  |
| Franco 2020 | RCT | Education | 12 weeks | Functional | Single leg stance eyes closed | Seconds | 4.9 | 3.4 | 35 | 2.3 | 1.8 | 36 | 2.6 | [1.33, 3.87] | P < 0.00001 | 1 | |  |
|  |  |  |  |  | Single leg balance eyes open | Seconds | 33.6 | 6.5 | 35 | 29.9 | 6.9 | 36 | 3.7 | [0.58, 6.82] | P = 0.02 | 1 | |  |
|  |  |  |  |  | Sit to stand (STS) | Seconds | 12.5 | 3.1 | 35 | 15.7 | 6.7 | 36 | -3.2 | [-5.62, -0.78] | P = 0.009 | 1 | |  |
|  |  |  |  |  | 4m walk time | Seconds | 3.8 | 0.9 | 35 | 4.3 | 1.1 | 36 | -0.5 | [-0.97, -0.03] | P = 0.04 | 0 | |  |
| Goldsmith 2021 | Cost effectiveness | No control | 56 weeks | Falls | No. of falls | Percentage | -52% | NR | NR | NR | NR | NR | NR | NR | NR | 1 | |  |
|  |  |  |  | Cost effectiveness | Cost savings | £ | £196m | NR | NR | NR | NR | NR | NR | NR | NR | 1 | |  |
| Hackney 2013 | Observational, pre-post | No control | 16 weeks | Functional | Chair stand test | Reps | 11.3 | 4 | 13 | No control | No control | No control | No control | No control | No control | 1 | |  |
| Hackney 2015 | QE | Exercise | 16 weeks | Functional | Berg Balance Scale (BBS) | Points | 49.6 | 3 | 14 | 49.6 | 4.6 | 18 | 0 | [-2.64, 2.64] | P = 1.00 | 0 | |  |
|  |  |  |  |  | Sensory organisation test (SOT) | Points | 62.6 | 12 | 14 | 60.3 | 14 | 18 | 2.3 | [-6.72, 11.32] | P = 0.62 | 1 | |  |
|  |  |  |  |  | 6-minute walk test (6MWT) | Meters | 333.3 | 115 | 14 | 317 | 134 | 18 | 16.3 | [-70.08, 102.68] | P = 0.71 | 1 | |  |
|  |  |  |  |  | Timed up and Go (TUG) | Seconds | 9.7 | 2.7 | 14 | 10.8 | 6.1 | 18 | -1.1 | [-4.25, 2.05] | P = 0.49 | 1 | |  |
|  |  |  |  | Quality of life | NEI VFQ–25 | Points | 53.6 | 27 | 14 | 56.3 | 21 | 18 | -2.7 | [-19.85, 14.45] | P = 0.76 | 0 | |  |
| Hamacher 2016 | RCT | Exercise | 24 weeks | Functional | Local dynamic stability | NA | NR | NR | 16 | NR | NR | 16 | NR | NR | NR | 1 | |  |
| Hofgaard 2019 | RCT | Usual care | 6 weeks | Functional | Berg Balance Scale (BBS) | Points | NR | NR | 15 | NR | NR | 10 | NR | NR | NR | 1 | |  |
|  |  |  |  |  | Fullerton Advanced Balance Scale (FAB) | Points | NR | NR | 15 | NR | NR | 10 | NR | NR | NR | 1 | |  |
|  |  |  |  |  | Timed up and go (TUG) | Seconds | NR | NR | 15 | NR | NR | 10 | NR | NR | NR | 0 | |  |
|  |  |  |  |  | Short Physical Performance Battery (SPPB) | Points | NR | NR | 15 | NR | NR | 10 | NR | NR | NR | 1 | |  |
|  |  |  |  |  | Chair stand test | Reps | NR | NR | 15 | NR | NR | 10 | NR | NR | NR | 1 | |  |
|  |  |  |  |  | 6-minute walk test (6MWT) | Meters | NR | NR | 15 | NR | NR | 10 | NR | NR | NR | 1 | |  |
| Kaewjoho 2020 | Observational, pre-post | No control | 6 weeks | Functional | Timed up and go (TUG) | Seconds | 9.08 | 1.1 | 61 | No control | No control | No control | No control | No control | No control | 1 | |  |
|  |  |  |  |  | Sit to stand (STS) | Seconds | 10.16 | 1.94 | 61 | No control | No control | No control | No control | No control | No control | 1 | |  |
|  |  |  |  |  | 6-minute walk test (6MWT) | Meters | 354.7 | 46.6 | 61 | No control | No control | No control | No control | No control | No control | 1 | |  |
|  |  |  | 24 weeks | Falls | No. of falls | Number | 4 | No data | 61 | No control | No control | No control | No control | No control | No control | 1 | |  |
| Kalyani 2020 | QE | Usual care | 12 weeks | Functional | Timed up and go (TUG) | Seconds | 10.6 | 2.9 | 17 | 11.4 | 3.3 | 16 | -0.8 | [-2.92, 1.32] | P = 0.46 | 1 | |  |
|  |  |  |  |  | Tinetti | Points | 25.53 | 2.69 | 17 | 21.94 | 6.05 | 16 | 3.59 | [0.36, 6.82] | P = 0.03 | 1 | |  |
|  |  |  |  |  | Berg Balance Scale (BBS) | Points | 50.76 | 5.17 | 17 | 47.5 | 9.06 | 16 | 3.26 | [-1.81, 8.33] | P = 0.21 | 1 | |  |
|  |  |  |  |  | Mini-BESTest (MBT) | Points | 20.24 | 5.34 | 17 | 17.5 | 6.61 | 16 | 2.74 | [-1.38, 6.86] | P = 0.19 | 1 | |  |
| Krampe 2010 | Observational, pre-post | No control | 6 weeks | Functional | Functional reach test | cm | NR | NR | NR | No control | No control | No control | No control | No control | No control | 1 | |  |
|  |  |  |  |  | Timed up and go (TUG) | Seconds | NR | NR | NR | No control | No control | No control | No control | No control | No control | 1 | |  |
| Kunkel 2017 | RCT | Usual care | 24 weeks | Functional | Berg Balance Scale (BBS) | Points | 50.4 | 5.3 | 31 | 52 | 4.5 | 15 | -1.6 | [-4.54, 1.34] | P = 0.29 | 0 | |  |
|  |  |  |  |  | Timed up and go (TUG) | Seconds | 13.8 | 4.4 | 31 | 12.3 | 3.5 | 15 | 1.5 | [-0.87, 3.87] | P = 0.21 | 0 | |  |
|  |  |  |  |  | 6-minute walk test (6MWT) | Meters | 367.3 | 100.5 | 31 | 393.2 | 90.1 | 15 | -25.9 | [-83.61, 31.81] | P = 0.38 | 0 | |  |
|  |  |  |  | Quality of life | PDQ39 | Points | 21.2 | 12.1 | 31 | 18.5 | 14.3 | 15 | 2.7 | [-5.70, 11.10] | P = 0.53 | 0 | |  |
| Leelapattana 2018 | RCT | Exercise | 12 weeks | Functional | Timed up and go (TUG) | Seconds | 8 | 1.5 | 19 | 10.1 | 3 | 20 | -2.1 | [-3.58, -0.62] | P = 0.005 | 1 | |  |
|  |  |  |  |  | Sit to stand (STS) | Seconds | 10.4 | 2.9 | 19 | 11.3 | 3.4 | 20 | -0.9 | [-2.88, 1.08] | P = 0.37 | 1 | |  |
| Li 2022 | RCT | Usual care | 12 weeks | Functional | Balance Check 636 Score rotational speed | Rad/s | 2.98 | 0.59 | 20 | 4.22 | 0.62 | 20 | -1.24 | [-1.62, -0.86] | P < 0.00001 | 1 | |  |
| Machacova 2017 | RCT | Usual care | 12 weeks | Functional | Get up and go test | Points | 8.62 | 2.37 | 92 | 8.39 | 2.2 | 97 | 0.23 | [-0.42, 0.88] | P = 0.49 | 1 | |  |
|  |  |  |  |  | Chair stand test | Reps | 10.27 | 4.2 | 27 | 7.2 | 3.55 | 25 | 3.07 | [0.96, 5.18] | P = 0.004 | 1 | |  |
|  |  |  |  |  | Sit and reach test | Cm | -1.37 | 7.36 | 27 | -8.48 | 11.04 | 25 | 7.11 | [1.97, 12.25] | P = 0.007 | 1 | |  |
|  |  |  |  | Strength | Arm curl test | Reps | 13.81 | 4.38 | 27 | 11.52 | 6.19 | 25 | 2.29 | [-0.65, 5.23] | P = 0.13 | 1 | |  |
| McKee 2013 | QE | Education | 12 weeks | Falls | Falls incidence | Number | 2 | NR | 24 | 0 | NR | 9 | RR 2.00 | [0.11, 38.08] | NR | 0 | |  |
|  |  |  |  | Functional | Timed up and go (TUG) | Seconds | 10.3 | 3.6 | 24 | 10.1 | 1.4 | 9 | 0.2 | [-1.51, 1.91] | P = 0.82 | 0 | |  |
|  |  |  |  | Quality of life | SF-12 Physical | Points | 41.3 | 12.6 | 24 | 40.8 | 11.5 | 9 | 0.5 | [-8.55, 9.55] | P = 0.91 | 1 | |  |
|  |  |  |  |  | SF-12 Mental | Points | 50 | 9.1 | 24 | 50.5 | 12.3 | 9 | -0.5 | [-9.32, 8.32] | P = 0.91 | 0 | |  |
|  |  |  |  |  | PDQ39 | Points | 18.7 | 9.4 | 24 | 19.3 | 8.1 | 9 | -0.6 | [-7.09, 5.89] | P = 0.86 | 0 | |  |
| McKinley 2008 | RCT | Exercise | 14 weeks | Functional | Sit to stand (STS) | Seconds | 10.67 | 4.41 | 14 | 11.32 | 5.76 | 11 | -0.65 | [-4.76, 3.46] | P = 0.76 | 1 | |  |
| Merom 2018 | RCT | Usual care | 52 weeks | Falls | No. of falls | Number | 257 | 1.03 | 275 | 187 | 0.8 | 247 | RR 1.23 | [1.14, 1.33] | P < 0.00001 | 0 | |  |
|  |  |  |  | Risk of falls | Physiological Performance Assessment (PPA) | Points | 1.02 | 1.43 | 275 | 0.69 | 1.23 | 249 | 0.33 | [0.10, 0.56] | P = 0.005 | 0 | |  |
|  |  |  |  | Functional | Short Physical Performance Battery (SPPB) | Points | 7.9 | 4.8 | 275 | 8.8 | 4.3 | 249 | -0.9 | [-1.68, -0.12] | P = 0.02 | 0 | |  |
|  |  |  |  |  | Chair stand test | Reps | 17.8 | 10.8 | 275 | 16.1 | 9.9 | 249 | 1.7 | [-0.07, 3.47] | P = 0.06 | 1 | |  |
|  |  |  |  | Quality of life | SF-12 Physical | Points | 39.8 | 10.9 | 275 | 40.8 | 10.8 | 249 | -1 | [-2.86, 0.86] | P = 0.29 | 0 | |  |
|  |  |  |  |  | SF-12 Mental | Points | 49.4 | 10.8 | 275 | 50.3 | 9.5 | 249 | -0.9 | [-2.64, 0.84] | P = 0.31 | 0 | |  |
| Noopud 2019 | RCT | Usual care | 12 weeks | Functional | Berg balance scale (BBS) | Points | 55 | NR | 22 | 50 | NR | 21 | NR | NR | NR | 1 | |  |
|  |  |  |  |  | Timed up and go (TUG) | Seconds | 6.26 | NR | 22 | 8.6 | NR | 21 | NR | NR | NR | 1 | |  |
|  |  |  |  |  | Sit to stand (STS) | Seconds | 0.32 | 0.25 | 22 | 0.69 | 0.23 | 21 | -0.37 | [-0.51, -0.23] | P < 0.00001 | 1 | |  |
| Nur 2022 | RCT | Usual care | 8 weeks | Risk of falls | Morse falls risk, no risk | Number | 8 | NR | 21 | 6 | NR | 20 | NR | NR | p < 0.05 | 1 | |  |
|  |  |  |  | Functional | Berg Balance Scale (BBS) independent | Number | 14 | NR | 21 | 9 | NR | 20 | NR | NR | NR | 0 | |  |
| Otoole 2015 | Mixed methods | No control | 6 weeks | Concern about falling | Falls efficacy scale-international (FES-I) | Points | 20 | NR | 35 | No control | No control | No control | No control | No control | No control | 1 | |  |
|  |  |  |  | Quality of life | EQ-VAS | Points | 85 | NR | 35 | No control | No control | No control | No control | No control | No control | 1 | |  |
| Pope 2019 | QE | Exercise | 8 weeks | Risk of falls | Physiological Performance Assessment (PPA) | Points | 1.2 | 1 | 50 | 0.4 | 0.9 | 89 | 0.8 | [0.47, 1.13] | P < 0.00001 | 0 | |  |
|  |  |  |  | Functional | Timed up and go (TUG) | Seconds | 7.8 | 2.7 | 50 | 7.7 | 2 | 89 | 0.1 | [-0.76, 0.96] | P = 0.82 | 0 | |  |
|  |  |  |  | Strength | Left leg strength |  | 0.27 | 0.1 | 50 | 0.28 | 0.09 | 89 | -0.01 | [-0.04, 0.02] | P = 0.56 | 0 | |  |
|  |  |  |  |  | Right leg strength |  | 0.26 | 0.11 | 50 | 0.28 | 0.1 | 89 | -0.02 | [-0.06, 0.02] | P = 0.29 | 0 | |  |
| Rawson 2019 | QE | Exercise | 12 weeks | Functional | Mini-BESTest (MBT) | Points | NR | NR | NR | NR | NR | NR | NR | NR | NR | 0 | |  |
| Rios 2015 | RCT | Usual care | 12 weeks | Functional | Timed up and go (TUG) | Seconds | 6.1 | 1.5 | 18 | 8 | 2.2 | 15 | -1.9 | [-3.21, -0.59] | P = 0.005 | 0 | |  |
|  |  |  |  |  | Mini-BESTest (MBT) | Points | 36.3 | 3 | 18 | 31.3 | 6.9 | 15 | 5 | [1.24, 8.76] | P = 0.009 | 1 | |  |
|  |  |  |  | Falls | No. of falls | Number | 2 | No data | 18 | 3 | No data | 15 | RR 0.56 | [0.11, 2.90] | P = 0.49 | 1 | |  |
| Rodrigues-Krause 2018 | RCT | Exercise | 8 weeks | Functional | Gait ability TUG | Meters/Second | 0.36 | NR | 10 | 0.39 | NR | 10 | NR | NR | NR | 0 | |  |
| Rodziewicz-Flis 2022 | RCT | Exercise | 12 weeks | Functional | 6-minute walk test (6MWT) | Meters | 419 | 8.5 | 10 | 422 | 11.4 | 10 | -3 | [-11.81, 5.81] | P = 0.43 | 1 | |  |
|  |  |  |  |  | Timed up and go (TUG) | Seconds | 6.5 | 0.23 | 10 | NR | NR | NR | NR | NR | NR | 1 | |  |
|  |  | Usual care |  |  | 6-minute walk test (6MWT) | Seconds | 419 | 8.5 | 10 | NR | NR | 10 | NR | NR | NR | 1 | |  |
|  |  |  |  |  | Timed up and go (TUG) | Meters | 6.5 | 0.23 | 10 | 10.33 | 0.62 | 10 | -3.83 | [-4.24, -3.42] | P < 0.00001 | 1 | |  |
| Shigematsu 2002 | QE | Usual care | 12 weeks | Functional | Single leg balance eyes open | Seconds | 24.6 | 17.3 | 20 | 16.6 | 15.8 | 18 | 8 | [-2.52, 18.52] | P = 0.14 | 0 | |  |
|  |  |  |  |  | Single leg balance eyes closed | Seconds | 4.1 | 2 | 20 | 5.3 | 5.3 | 18 | -1.2 | [-3.80, 1.40] | P = 0.37 | 1 | |  |
|  |  |  |  |  | Functional reach test | Cm | 26.1 | 3.6 | 20 | 23.3 | 7.6 | 18 | 2.42 | [1.65, 3.19] | P < 0.00001 | 0 | |  |
|  |  |  |  | Strength | Keeping a half squat position | Seconds | 30.8 | 17.7 | 20 | 34.9 | 20.4 | 18 | -4.1 | [-16.31, 8.11] | P = 0.51 | 1 | |  |
|  |  |  |  |  | Hand grip strength | Kg | 21.4 | 2.9 | 20 | 19.5 | 3.6 | 18 | 1.9 | [-0.19, 3.99] | P = 0.08 | 1 | |  |
| Sohn 2018 | Observational, pre-post | No control | 15 weeks | Functional | Walking balance | Cm2 | 38.2 | 18.2 | 15 | No control | No control | No control | No control | No control | No control | 1 | |  |
|  |  |  |  |  | Standing balance, COP distance | Cm | 84.2 | 34.4 | 15 | No control | No control | No control | No control | No control | No control | 1 | |  |
| Tillman 2020 | QE | Usual care | 12 weeks | Functional | Berg Balance Scale (BBS) | Points | 54 | 8.75 | 10 | 37 | 4.6 | 10 | 17 | [10.87, 23.13] | P < 0.00001 | 0 | |  |
|  |  |  |  | Quality of life | PDQ39 | Points | 49 | 27.9 | 10 | 66.5 | 9.3 | 10 | -17.5 | [-35.73, 0.73] | P = 0.06 | 0 | |  |
| Vella-Burrows 2021 | Mixed methods | No control | 24 weeks | Quality of life | EQ-5D-5L | Points | 75.93 | 15.05 | 43 | No control | No control | No control | No control | No control | No control | 1 | |  |
|  |  |  |  |  | Geriatric Depression Scale (GDS) | Points | 2.3 | 1.4 | 8 | 5.8 | 2.4 | 6 | -3.5 | [-5.65, -1.35] | P = 0.001 | 1 | |  |
|  |  |  |  |  | PDQ39 | Points | 16.5 | 12.7 | 8 | 51.7 | 24.9 | 7 | -35.2 | [-55.64, -14.76] | P = 0.0007 | 1 | |  |
| Ventura 2016 | QE | Usual care | 20 weeks | Concern about falling | Falls efficacy scale-international (FES-I) | Points | 20.4 | 4.5 | 8 | 36.4 | 10.5 | 7 | -16 | [-24.38, -7.62] | P = 0.0002 | 1 | |  |
|  |  |  |  | Functional | Timed up and go (TUG) | Seconds | 11.3 | 1.9 | 8 | 16.3 | 6.5 | 6 | -5 | [-10.37, 0.37] | P = 0.07 | 0 | |  |
|  |  |  |  |  | Standing balance test | Points | 21.8 | 10.4 | 7 | 10.5 | 10.8 | 5 | 11.3 | [-0.91, 23.51] | P = 0.07 | 1 | |  |
| Wang 2021 | RCT | Education | 12 weeks | Functional | Sit to stand (STS) | Seconds | 6.44 | 1.28 | 22 | 7.05 | 1.19 | 22 | -0.61 | [-1.34, 0.12] | P < 0.00001 | 0 | |  |
| Weighart 2020 | QE | Usual care | 10 weeks | Functional | Center of pressure (CoP) 30s area eyes open | Mm2 | 0.05 | 0.02 | 11 | 0.05 | 0.02 | 6 | 0 | [-0.02, 0.02] | P = 1.00 | 0 | |  |
|  |  |  |  |  | Center of pressure (CoP) 30s area eyes closed | Mm2 | 0.07 | 0.03 | 11 | 0.09 | 0.14 | 6 | -0.02 | [-0.13, 0.09] | P = 0.73 | 0 | |  |

*****Highlighted in yellow – Lower scores indicate improvement, NR – not reported in the study; in red text – not significant
